# Far-UVC 222 nm Treatment: Effects of Nitrate/Nitrite on Disinfection Byproduct Formation Potential

**DOI:** 10.1021/acs.est.4c04258

**Published:** 2024-08-12

**Authors:** Jiale Xu, Ryan J. Kann, Dauda Mohammed, Ching-Hua Huang

**Affiliations:** † Department of Civil, Construction and Environmental Engineering, North Dakota State University, Fargo, North Dakota 58102, United States; ‡ School of Civil and Environmental Engineering, Georgia Institute of Technology, Atlanta, Georgia 30332, United States; § School of Biological Sciences, Georgia Institute of Technology, Atlanta, Georgia 30332, United States

**Keywords:** KrCl* excimer lamp, far-UVC 222 nm, disinfection byproducts, nitrate/nitrite, chloropicrin, nitration, water treatment

## Abstract

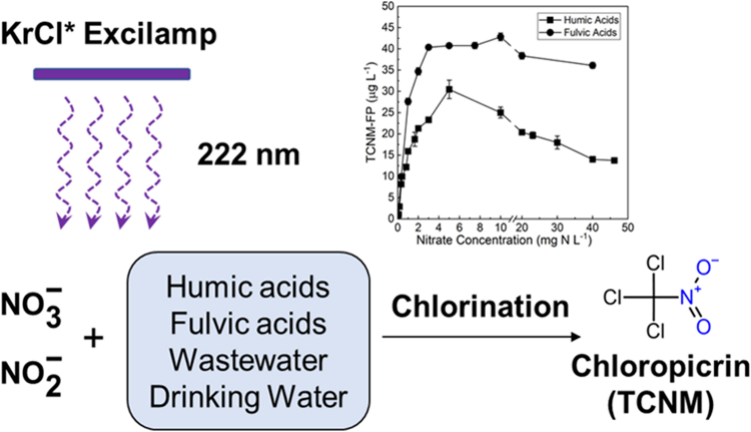

Irradiation at far ultraviolet C (far-UVC) 222 nm by krypton chloride (KrCl*) excilamps can enhance microbial disinfection and micropollutant photolysis/oxidation. However, nitrate/nitrite, which absorbs strongly at 222 nm, may affect the formation of disinfection byproducts (DBPs). Herein, we evaluated model organic matter and real water samples and observed a substantial increase in the formation potential for trichloronitromethane (chloropicrin) (TCNM-FP), a nitrogenous DBP, by nitrate or nitrite after irradiation at 222 nm. At a disinfection dose of 100 mJ·cm^–2^, TCNM-FP of humic acids and fulvic acids increased from ∼0.4 to 25 and 43 μg·L^–1^, respectively, by the presence of 10 mg-N·L^–1^ nitrate. For the effect of nitrate concentration, the TCNM-FP peak was observed at 5–10 mg-N·L^–1^. Stronger fluence caused a greater increase of TCNM-FP. Similarly, the increase of TCNM-FP was also observed for wastewater and drinking water samples containing nitrate. Pretreatment using ozonation and coagulation, flocculation, and filtration or the addition of H_2_O_2_ can effectively control TCNM-FP. The formation potential of other DBPs was minorly affected by irradiation at 222 nm regardless of whether nitrate/nitrite was present. Overall, far-UVC 222 nm treatment poses the risk of increasing TCNM-FP of waters containing nitrate or nitrite at environmentally relevant concentrations and the mitigation strategies merit further research.

## Abstract 2

Far-UVC 222 nm treatment poses the risk of increasing the formation potential of chloropicrin for nitrate/nitrite-containing waters and should consider mitigation strategies.

## 1 Introduction

Krypton chloride (KrCl*) excimer lamps (excilamps) emitting light in the far ultraviolet C (far-UVC) region at 222 nm have emerged as a promising water treatment technology.^1−11^ KrCl* excilamps enhanced the inactivation over the conventional low-pressure mercury UV lamps (LPUV, wavelength at 254 nm) for bacteria (e.g., *Escherichia coli* and *Bacillus subtilis*)^7−13^ and viruses (e.g., MS2, adenoviruses, and SARS-CoV-2).^4−9,14,15^ The lower wavelength also holds the potential to efficiently remove organic micropollutants (OMPs) by direct photolysis^16,17^ or advanced oxidation processes (AOPs).^18−23^ A recent survey of 46 OMPs showed that the fluence-normalized direct photolysis rate was over 10 times greater at 222 nm than at 254 nm for 31 compounds, regardless of whether the OMPs absorbed light more strongly or weakly at 222 nm.^16^ The lower wavelength at 222 nm overlaps with the light absorption of common inorganic oxidants better than 254 nm. Hence, combining KrCl* excilamps with hydrogen peroxide, free chlorine, and peroxydisulfate, respectively, enhanced the removal of OMPs more significantly than LPUV lamps at the same fluence basis, due to stronger activation of oxidants and higher production of reactive radical species under the AOP.^18−23^ Moreover, KrCl* excilamps feature minimal adverse effects on human skin and eyes,^24−26^ thermal stability at low temperatures at around 5–10 °C,^27^ and the absence of toxic mercury.

Meanwhile, the impacts of water matrix constituents play a critical role in the UV processes due to potential effects of light screening, reactive species quenching, and sensitizing. Compared with LPUV, the 222 nm-based processes are expected to be impacted more strongly by water matrix constituents, particularly nitrate and nitrite.^16,28^ Among the common water constituents at their environmentally relevant concentrations, nitrate and nitrite are the dominant species in screening light at 222 nm, owing to their 916 and 292 times higher molar absorption coefficients at 222 nm than at 254 nm, respectively.^16^ Nitrate at 0.5–10 mg-N·L^–1^ was reported to significantly inhibit *E. coli* inactivation at 222 nm by 1.1–2.7 times, primarily due to its strong light screening effect, while negligible effects were observed at 254 nm.^28^ Further, the photolysis of nitrate and nitrite can generate hydroxyl radical (^•^OH) and reactive nitrogen species (RNS; e.g., ^•^NO_2_, N_2_O_4_, ^•^NO, and ONOO^–^) through reactions 1 to 8(29)
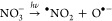
1
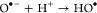
2
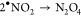
3
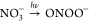
4
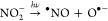
5
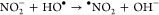
6
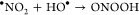
7
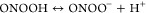
8

Besides the strong absorption at 222 nm for nitrate/nitrite, the quantum yield (Φ) of RNS was also higher at lower wavelengths, suggesting the generation of more RNS by KrCl* excilamps than LPUV.^30^ For example, the Φ of photolysis of nitrate (reactions 1 and 4) was 2.5 and 2.8 times higher, respectively, at 220 nm than at 254 nm.^30^ RNS formed in nitrate/nitrite photolysis can react with organic compounds to form nitro- or nitroso compounds, which was tested to be stronger at 222 nm than at 254 nm by a recent study using phenol as the precursor.^28^

The presence of nitrate/nitrite in UV and sunlight processes poses a risk to increase the formation potential of nitrogenous disinfection byproducts (N-DBPs),^31−33^ a group of compounds that are 100–1000 times more cytotoxic and genotoxic than the regulated trihalomethane and haloacetic acid DBPs by the United State Environmental Protection Agency (U.S. EPA).^34^ The reason is that the nitrating agents (e.g., ^•^NO_2_, N_2_O_4_, and ONOO^–^) generated from nitrate/nitrite photolysis can react with organic matter in water to form nitrogenous compounds, potent precursors of N-DBPs.^31−33^ Previous research found that the treatment by medium pressure UV (MPUV) followed by chlorination greatly increased the formation of trichloronitromethane (TCNM or chloropicrin: a N-DBP) in waters with elevated nitrate/nitrite concentrations.^31,33^ After pretreatment of humic acids in the presence of 3 mg-N·L^–1^ nitrate by 280 mJ·cm^–2^ MPUV exposure, TCNM formation by chlorination increased by seven times compared with that without UV pretreatment.^31^ Considering the high light absorption and quantum yield for nitrate and nitrite at 222 nm,^16^ KrCl* excilamp treatment is expected to exert a greater risk in increasing TCNM formation than LPUV and MPUV. However, nitrate photolysis at 222 nm also enables in situ AOP by generating ^•^OH,^20^ which can oxidize and remove DBP precursors. Overall, it still remains unclear how the net outcome of nitrate/nitrite photolysis at 222 nm affects the formation potential of TCNM and other DBPs.

This study aimed to improve our understanding of the effects of nitrate and nitrite on the formation potential (FP) of DBPs in water treated by KrCl* excilamp irradiation. Humic acids (HA) and fulvic acids (FA) in water were first examined as model NOM for the effects of nitrate/nitrite concentration and 222 nm irradiation fluence on the formation potential of TCNM and other nine DBPs (including trihalomethanes (THMs), haloacetonitriles (HANs), and haloketones (HKs)). The DBP-FP of water samples under chlorination was quantified. Then, similar experiments were conducted using real wastewater and drinking water samples, and the impacts of different drinking water treatment schemes on the results were compared. Lastly, the control of DBP-FP using hydrogen peroxide advanced oxidation was evaluated in the presence of nitrate as a promising mitigation strategy.

## 2 Materials and Methods

### 2.1 Materials

Detailed information about the chemicals is provided in Text S1.

### 2.2 Irradiation Experiments Using Model Compounds

Humic acids (HA, MP Biomedicals) and fulvic acids (FA, Suwannee River FA from International Humic Substances Society) were used as model NOM to evaluate the effects of nitrate/nitrite on DBP-FP. A bench-scale collimated beam apparatus (Figure S1a) with a filtered Ushio KrCl* excilamp emitting narrowly at 222 nm (Figure S1b) was used in this study. Reaction solution (70 mL) of HA or FA (3.5 mg-C·L^–1^) in deionized water, buffered at pH 6.8 by 10 mM phosphate, and spiked with 100 mg·L^–1^ chloride, 0.1 mg·L^–1^ bromide, and a desired amount of nitrate or nitrite, was placed in a crystallizing dish and irradiated under the KrCl* excilamp at room temperature (25 °C). The emphasis of this study was on nitrate, because of its much higher concentration than nitrite in most environmental waters. Nitrate was spiked at 0.1–46 mg-N·L^–1^ to simulate low to high levels of contamination. The impact of nitrite was studied in HA solutions spiked with 0.2–1.4 mg-N·L^–1^ nitrite. All of the solutions were stirred continuously at 300 rpm. The averaged fluence rate was 1.7 mW·cm^–2^ (31.5 μEinstein·m^–2^·s^–1^), measured by iodide-iodate actinometry according to procedures previously described.^16^ The distance between the lamp and the surface of the solutions was about 5.5 cm. The effective path length was measured as 1.8 cm. A UV fluence of 100 mJ·cm^–2^ was applied for most experiments except otherwise specified. To assess the effect of UV fluence, UV doses of 0–1000 mJ·cm^–2^ were conducted to cover conditions from typical disinfection to AOPs, using HA solutions spiked with 10 mg-N·L^–1^ nitrate. All experiments were conducted in triplicate. After irradiation, water samples were analyzed for DBP-FP by chlorination using procedures described in Texts S2 and S3. For samples spiked with nitrite, a higher dose of chlorine was used, as described in Text S2 to account for extra chlorine consumption by nitrite.

### 2.3 Irradiation Experiments Using Wastewater and Drinking Water Samples

The impact of nitrate was evaluated in four real water samples using the same setup as that described in Section 2.2. A nitrified secondary wastewater effluent sample (labeled as “WW”) was collected from a municipal wastewater treatment plant (WWTP). Three drinking water samples were taken from a local drinking water treatment plant (DWTP) at source water, after ozonation, and after coagulation/flocculation/filtration, which were labeled as “DW Raw Water”, “DW After Ozone”, and “DW After Filter”, respectively. All four samples were shipped on ice to the laboratory within 2 h, filtered immediately upon receipt by precombusted 0.7 μm glass fiber filters, and stored at 5 °C before use. Figure S2 shows the treatment schemes of WWTP and DWTP, and Table S1 shows the water quality of each sample.

Three tiers of experiments were conducted. (1) Original samples after filtration were first tested to mimic the direct application of KrCl* excilamps at WWTP or DWTP. (2) Samples were spiked with varying concentrations of nitrate to evaluate its impact on DBP-FP. Note that the WW sample had a high nitrate concentration at 15.76 mg-N·L^–1^ and thus was diluted first to a low concentration at 1.7 mg-N·L^–1^. (3) To compare the properties of organic matter in the real water samples that could be transformed to TCNM precursors, samples were diluted to achieve the same total organic carbon (TOC) concentration of 3.5 mg-C·L^–1^. Then, the initial nitrate concentration was controlled at 0.33 or 10 mg-N·L^–1^.

### 2.4 Control of TCNM Precursors by H_2_O_2_

To control the formation of TCNM precursors from nitrate in KrCl* excilamp irradiation, H_2_O_2_ was added to HA solutions (3.5 mg-C·L^–1^) with 10 mg-N·L^–1^ nitrate before irradiation. The impacts of the H_2_O_2_ dose at 100–1000 μM and UV fluence at 200–2000 mJ·cm^–1^ were evaluated. After irradiation, the residual H_2_O_2_ was quenched by sodium thiosulfate based on stoichiometry, and then the sample was evaluated for DBP-FP. Unquenched samples were also collected to measure H_2_O_2_ concentration by the 2,2′-azino-bis(3-ethylbenzothiazoline-6-sulfonic)acid (ABTS) method.^35^ Briefly, fresh ABTS stock solution was prepared in 0.2 mM buffer at pH 7 using 100 mM phosphate. Samples containing H_2_O_2_ (1.0 mL) were mixed with 9.0 mL of ABTS solution and 0.15 mL of horseradish peroxidase at 0.5 units per mL, and the concentration of ABTS^•+^ generated from H_2_O_2_ was immediately measured by a UV–visible spectrophotometer at 405 nm.

## 3 Results and Discussion

### 3.1 Effects of Nitrate and Nitrite in Model NOM Systems

#### 3.1.1 Effect of Nitrate on TCNM-FP

**Figure 1:**
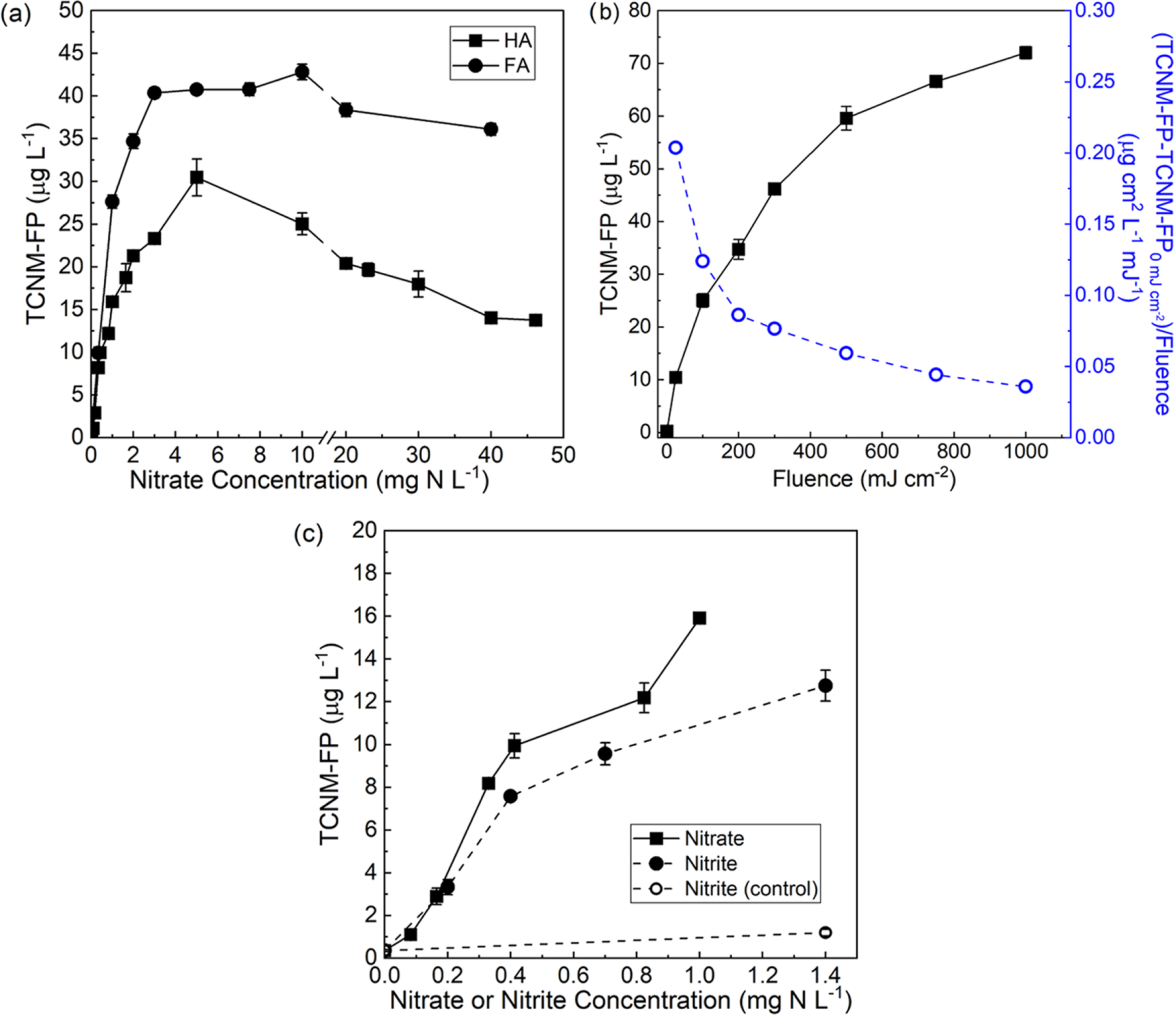
Formation potential of TCNM (TCNM-FP) for solutions with model HA or FA in the presence of nitrate or nitrite after 222 nm irradiation: (a) impact of nitrate concentration for HA and FA, (b) varying UV fluences for HA, and (c) varying concentrations of nitrite for HA as compared with nitrate. Conditions: 3.5 mg-C·L^–1^ HA or FA at pH 6.8 buffered by 10 mM phosphate, 100 mg·L^–1^ chloride, 0.1 mg·L^–1^ bromide, 100 mJ·cm^–2^ UV fluence for (a, c), and 10 mg-N·L^–1^ nitrate for (b). In (c), nitrate data are reproduced from (a), and open circle dots represent dark control without irradiation.

Effects of nitrate/nitrite on TCNM-FP for real water samples were first assessed using two selected model organic matters, HA and FA because they are major organic constituents in surface water and wastewater.^36^Figure 1a shows that the presence of nitrate (0–46 mg-N·L^–1^) caused the increase of TCNM-FP of both HA and FA after 222 nm irradiation at 100 mJ·cm^–2^, which was in the range of UV disinfection dose for water treatment. At 10 mg-N·L^–1^ nitrate, TCNM was increased by 71–119 times from 0.35 to 25–43 μg·L^–1^ for HA and FA, compared with those in the absence of nitrate. The effect was dependent on nitrate concentration. When the nitrate concentration was less than 5 mg-N·L^–1^, the 222 nm irradiation increased TCNM-FP from 0.35 to 30.5 μg·L^–1^ for HA and from 0.36 to 40.7 μg·L^–1^ for FA. When the nitrate concentration was in the range of 5–46 mg-N·L^–1^, no further significant increase in TCNM-FP was observed. Instead, TCNM-FP either decreased by 55% to 13.8 μg·L^–1^ for HA, or slightly increased and then decreased to 36.1 μg·L^–1^ for FA. The maximum TCNM-FP occurred at around 3–10 mg-N·L^–1^ of nitrate, a common condition for water samples, suggesting a high potential of this risk in realistic scenarios. Comparison between different organic matter showed a stronger increase for FA than for HA at all tested nitrate concentrations. For example, at 10 mg-N·L^–1^ nitrate, TCNM-FP of FA was 42.8 μg·L^–1^, 1.7 times of that for HA. This phenomenon may be attributed to that FA features more abundant phenolic moieties, the compounds that are prone to be nitrated by nitrating agents^37−39^ to form TCNM precursors,^32^ than HA.^40−42^ It should be acknowledged that results from HA and FA only provide a potential risk and cannot be directly applied to drinking water or wastewater samples because HA and FA do not represent other organics such as hydrophilic substances in environmental samples. Hence, real water samples were also tested in this study, as shown in Section 3.2.

Previous research has shown that the increase of TCNM-FP by ^•^NO_2_ (nitrogen dioxide radical, the dominant nitrating agent)^31^ for nitrate-contaminated waters in UV-chlorination combined processes commonly follows the steps of: (1) ^•^NO_2_ formation by nitrate photolysis, (2) excitation of organics to become organic radicals, (3) formation of TCNM precursors through radical reactions between ^•^NO_2_ and organic radicals, and (4) TCNM formation by chlorination (see scheme in Figure S4).^43,44^ For example, photonitration of phenol follows reactions 9 and 10(43)
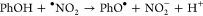
9
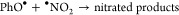
10

The formation of phenolic radical can also occur by direct photolysis reaction 11(45) and oxidation by hydroxyl radical (^•^OH) reaction 12(46)
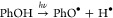
11
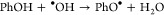
12

Nitrating agents, such as ^•^NO_2_ and N_2_O_4_, can also hydrolyze through reactions 3 and 13 to form nitrate and nitrite.
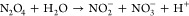
13

The decrease of TCNM-FP at high nitrate concentrations (Figure 1a) may be caused by three reasons: (1) inhibited formation of ^•^NO_2_, (2) inhibited formation of organic radical, and (3) chlorine quenching by formed nitrite. First, the steady-state concentrations of ^•^NO_2_ ([^•^NO_2_]_ss_) were estimated using probe compounds, 4-chlorophenol and nitrobenzene, as described in Text S4. The presence of 40 mg-N·L^–1^ nitrate caused a 53% decrease of [^•^NO_2_]_ss_ for both FA and HA solutions compared to that at 5 mg-N·L^–1^ nitrate (Figure S5a), suggesting that the formation or decay of ^•^NO_2_ was affected by high nitrate concentrations. Two possibilities were hypothesized for this impact. The first possibility was that extra nitrate reacted with ^•^NO_2_. To test this hypothesis, NO_2_ gas at 100 ppm in nitrogen gas was purged through 4-chlorophenol solutions at different nitrate concentrations (0–40 mg-N·L^–1^) as explained in Text S5. The formation rates of 4-chloro-2-nitrophenol and nitrite did not change upon a change of nitrate concentration (Figure S6), indicating that the reaction between nitrate and ^•^NO_2_ should be minor. The second possibility was that the formation of nitrating agents was shifted from ^•^NO_2_ to weaker nitrating agents at high nitrate concentrations. Nitrate photolysis can also produce other RNS, such as peroxynitrite (ONOO^–^), that features more than 100 times lower second-order rate constants than ^•^NO_2_ for most organics.^100^ Thus, a favorable formation of peroxynitrite over ^•^NO_2_ would result in a decreased level of nitration of organic matter. To test this hypothesis, phenol was selected as a probe to assess the change of nitrating agents caused by elevated nitrate concentrations. We found that the decay rate of phenol by other RNS was indeed higher at 40 mg-N·L^–1^ than at 5 mg-N·L^–1^ (Figure S5b), supporting the idea that the formation of other RNS was enhanced in the presence of a substantial amount of nitrate. This shift from ^•^NO_2_ to other RNS likely played an important role in the decrease of TCNM-FP at high nitrate concentrations. Nevertheless, nitrate photolysis under UV irradiation is quite complicated, and further research on the detailed mechanism of the impacts of nitrate concentration on photolysis pathways is warranted.

The second reason to decrease TCNM-FP was that the formation of organic radicals through direct photolysis and the ^•^OH reaction could be inhibited by high nitrate concentrations. Previous research reported that hydrogen abstraction of aniline by ^•^OH can form an anilino radical (C_6_H_5_NH^•^) to react with ^•^NO_2_ yielding nitrated aniline.^47^ To evaluate this process for organic matter, *tert*-butyl alcohol (TBA) was employed as a quencher to assess the role of ^•^OH in nitration (details are described in Text S6). As shown in Figure S5, the estimated steady-state concentration of ^•^OH ([^•^OH]_ss_) was decreased significantly by the spike of TBA, while ^•^NO_2_ and other RNS were not greatly impacted, suggesting that quenching ^•^OH potentially does not affect nitrating agents in nitrate photolysis. For TCNM-FP, the presence of 5 mM TBA resulted in a 14–17% decrease in TCNM-FP (Figure S7), indicating that ^•^OH contributed to the nitration of organics, potentially via enhanced formation of organic radicals. However, when the nitrate concentration was increased from 5 to 40 mg-N·L^–1^, [^•^OH]_ss_ increased by 40% for HA or changed little for FA, indicating that the formation of organic radicals via ^•^OH was not inhibited by the increased nitrate concentration. As another pathway to form organic radicals, direct photolysis of organics was potentially suppressed by nitrate owing to light screening. As shown in Figure S8b, light absorbance by HA through the water depth (1.8 cm) decreased from 0.707 in the absence of nitrate to 0.036 at 40 mg-N·L^–1^ nitrate. Hence, photonitration of organics (reaction 10) was likely restricted by the limited formation of organic radicals in reaction 11.

The third reason to decrease TCNM-FP was that during the FP test chlorine could be quenched by nitrite formed from nitrate photolysis. However, limited amounts of nitrite (0.05–0.17 mg-N·L^–1^) were observed after irradiation, and a minor change in nitrite concentration was induced by the increase of nitrate concentration (Figure S9). Further, abundant chlorine residual at around 5–6 mg-Cl_2_·L^–1^ remained after the FP test (Figure S3), suggesting sufficient chlorine was available for TCNM formation.

Overall, in the far-UVC (222 nm) treatment of NOM-containing water, the presence of nitrate up to 10 mg-N·L^–1^ significantly enhanced TCNM-FP. Higher nitrate concentrations decreased TCNM-FP by shifting to the formation of weaker nitrating agents as well as inhibiting the formation of organic radicals via light screening.

The effect of 222 nm UV fluence on the increase of TCNM-FP by nitrate (at 10 mg-N·L^–1^) was also assessed (Figure 1b). It was found that the higher the UV fluence, the higher the resulted TCNM-FP. TCNM-FP reached 72 μg·L^–1^ at UV fluence of 1000 mJ·cm^–2^. Interestingly, the increased TCNM-FP normalized by fluence was lower at a higher fluence, which can be attributed to greater nitrite formation to consume chlorine in the FP test. As fluence was increased, nitrite concentration increased from 0 to 0.96 mg-N·L^–1^ (Figure S9), which can consume a large amount of chlorine. To further confirm this effect, nitrite was spiked into samples exposed to 100 mJ·cm^–2^ fluence to reach the same nitrite level as those after 1000 mJ·cm^–2^ fluence prior to the FP Test. Indeed, the level of TCNM-FP decreased by 25% due to chlorine quenching by nitrite (Figure S10). Further, a higher chlorine dose was used for the FP test for samples after 1000 mJ·cm^–2^ irradiation to account for the extra nitrite, and an increase of TCNM-FP by 19% was observed, confirming available TCNM precursors. Without nitrate, UV 222 nm alone did not exert big impact on TCNM-FP. Figure S11 shows that TCNM-FP only slightly increased from 0.29–0.34 to 0.40–0.79 μg·L^–1^ when fluence was increased from 0 to 1000 mJ·cm^–2^.

#### 3.1.2 Effect of Nitrate on FP of Other DBPs

For the other nine DBPs including THMs, HANs, and HKs, neither UV 222 nm alone (Figure S12) nor nitrate combined with UV 222 nm (Figure S13) exhibited a strong impact on their FP with the change mostly less than 20% except for 1,1-dichloropropanone (1,1-DCP) and bromoform (TBM). UV 222 nm combined with nitrate can enable in situ AOP by generating ^•^OH at a higher steady-state concentration than UV_254_/H_2_O_2_, a conventional AOP.^20^ Our results suggested that AOP using UV_222_/nitrate had little impact on the FP of HA and FA for most of the nine tested DBPs. Previous studies have shown that the effect of UV_254_/H_2_O_2_ on THM-FP and HAA-FP was a function of numerous parameters and highly dependent on water quality.^48−51^ Thus, our conclusion should be evaluated further in the future using more water samples. As the exception, 1,1-DCP-FP from HA decreased by 69% with the increase of nitrate from 0 to 46 mg-N·L^–1^ after 100 mJ·cm^–2^ irradiation, and decreased by 92% with the increase of fluence from 0 to 1000 mJ·cm^–2^ in the presence of 10 mg-N·L^–1^ nitrate. Similar results for the impact of nitrate were also observed for FA (Figure S13f). The decrease in the level of 1,1-DCP-FP is likely attributed to two reasons. First, the precursors of 1,1-DCP can be removed by oxidation with ^•^OH. A previous study reported that the treatment by vacuum UV (VUV) decreased the formation of 1,1-DCP from naproxen primarily due to the formation of ^•^OH from water by VUV.^52^ The same study also observed minimal change on the formation of other DBPs, such as 1,1,3-trichloropropanone (1,1,3-TCP) and trichloroacetic acid (TCAA).^52^ Further evaluation of the role of ^•^OH on 1,1-DCP-FP is discussed in Section 3.3. Second, the precursors of 1,1-DCP may be converted to TCNM precursors by nitrate and UV 222 nm. For TBM, high fluence caused the decrease of FP, which may also be caused by the above-mentioned two reasons. However, these two preliminary hypotheses require future validation.

#### 3.1.3 Effect of Nitrite on FP of TCNM and Other DBPs

Figure 1c shows that nitrite also increased the TCNM-FP of HA after irradiation at 222 nm. Higher nitrite concentration led to higher TCNM-FP. At 1.4 mg-N·L^–1^ nitrite, TCNM-FP was 12.8 μg·L^–1^, 36 times of that in the absence of nitrite. Like nitrate, the risk in increasing TCNM-FP occurred for nitrite even at concentrations less than the EPA regulation of 1 mg-N·L^–1^. Besides photonitration, nitrite can react with chlorine in FP to form a nitrating agent ClNO_2_ to increase TCNM.^31^ Hence, dark control experiments at 1.4 mg-N·L^–1^ nitrite were conducted and showed only 9% (i.e., 1.2 μg·L^–1^) TCNM-FP of that after irradiation (Figure 1c), suggesting that the ClNO_2_ pathway was minor. Comparison between nitrate and nitrite showed that nitrite generally exhibited a weaker effect than nitrate. TCNM-FP in the presence of 0.4 mg of N·L^–1^ nitrite was 76% of that spiked with the same concentration of nitrate. Nitrite (ε_222_ = 3507 M^–1^·cm^–1^) features 28% times higher molar absorption coefficient than nitrate (ε_222_ = 2747 M^–1^·cm^–1^).^16^ However, the formation of nitrating agents is more complex for nitrite than for nitrate. Nitrate photolysis directly forms ^•^NO_2_reaction 1 or ONOO^–^reaction 4 in one step, while nitrite requires two steps, nitrite photolysis reaction 5 and oxidation of nitrite reaction 6 by ^•^OH to generate nitrating agents. Moreover, the quantum yield (Φ_nitrite_ = 0.046)^29^ of nitrite photolysis was 3 times less than the combined quantum yield of reactions 1 and 4 for nitrate photolysis (Φ_nitrate_ = 0.139)^30^ at 254 nm. Assuming that photolysis of nitrate and nitrite at 222 nm followed a similar trend at 254 nm, it is expected that nitrate photolysis still generates higher concentrations of nitrating agents than nitrite. Additionally, ^•^OH produced from nitrite photolysis can be quenched by organic matter, e.g., HA and FA, inhibiting the reaction between nitrite and ^•^OH to form nitrating agents reaction 6. However, the quenching of ^•^OH is expected to not highly affect the formation of nitrating agents from direct photolysis of nitrate reactions 1 and 4. For other DBPs, minimal changes in FP were observed by nitrite and 222 nm irradiation (Figure S14). Because of the low concentration of nitrite in the environment and its minor effects on DBP-FP, nitrate was selected as the focus for the WW and DW samples in Section 3.2.

### 3.2 Effects of Nitrate on DBP-FP of WW and DW Samples

#### 3.2.1 Real Water Samples

**Figure 2:**
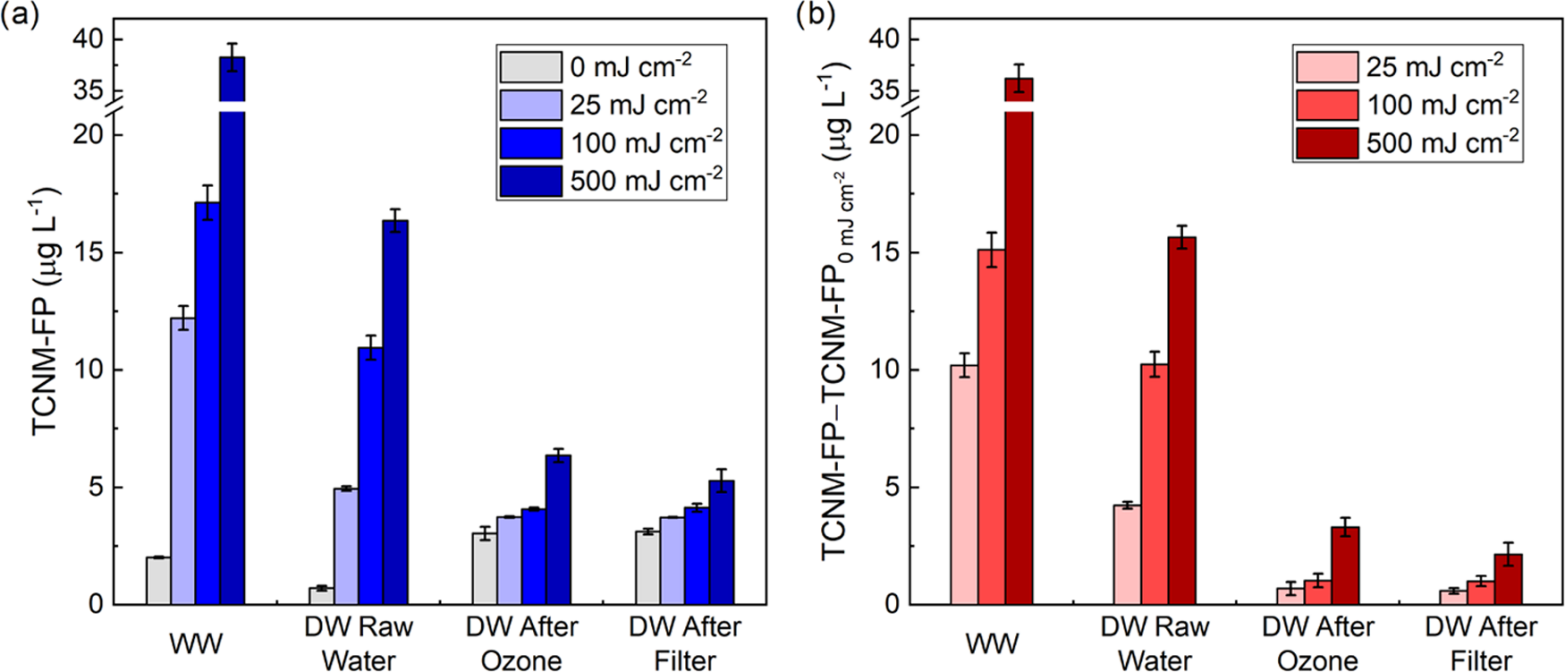
(a) Absolute formation potential of TCNM (TCNM-FP) and (b) relative TCNM-FP to nonirradiated condition for wastewater and drinking water samples after 222 nm irradiation at varying fluences.

As representatives of environmental waters, WW and DW samples without any spikes of nitrate were first studied to evaluate the effect of nitrate on DBP-FP during treatment by KrCl* excilamp irradiation. As shown in Figure 2a, 222 nm irradiation increased TCNM-FP of real water samples, and the higher irradiation dose rendered a greater increase, which were similar to the results from HA and FA. At 25 mJ·cm^–2^, a UV dose common for disinfection in engineered systems, TCNM-FP was increased by 10.2, 4.2, and 0.7–0.6 μg·L^–1^ for WW sample, DW raw water, and DW after pretreatment (Figure 2b), respectively. An AOP dose of 500 mJ·cm^–2^ further increased TCNM-FP. Figure 2b also shows that the increase of TCNM-FP for WW was nearly twice that for DW raw water, which was likely caused by the much higher nitrate concentration (15.8 mg-N·L^–1^) in WW than in DW (∼0.33 mg-N·L^–1^). For DW, the increase of TCNM-FP for “DW After Ozonation” was 10%–21% of that for “DW Raw Water” for all three fluences (25, 100, and 500 mJ·cm^–2^). Pretreatment by ozonation removed 14% of TOC (0.7 mg-C·L^–1^) (Table S1), and the moieties that were reactive to conversion to TCNM precursors were potentially removed to a large extent (see details in Section 3.2.3). Additional treatment by conventional coagulation/flocculation/filtration on ozonated DW further lowered TCNM-FP by 2% to 35%, likely due to the removal of precursors as indicated by 10% decrease of TOC (Table S1). For samples without irradiation (Figure 2a), WW exhibited 2.8 times higher TCNM-FP than “DW Raw Water”, suggesting that TCNM precursors were more abundant in municipal wastewater than in surface water, which is consistent with results in previous studies.^32,53^ Compared to “DW Raw Water”, pretreatment by ozonation increased TCNM-FP from 0.7 to 3.0 μg·L^–1^, likely owing to the oxidation of amines in dissolve organic matter to nitro compounds, the precursors of TCNM.^54−56^ This phenomenon has been widely observed in wastewater recycling processes^57^ and drinking water treatment.^58^ Coagulation/flocculation/filtration did not change TCNM-FP for preozonated samples, potentially because TCNM precursors after ozonation were small and hydrophilic,^59^ which were challenging to be removed by coagulation/flocculation/filtration.^60^

#### 3.2.2 Effects of Nitrate Concentrations

**Figure 3:**
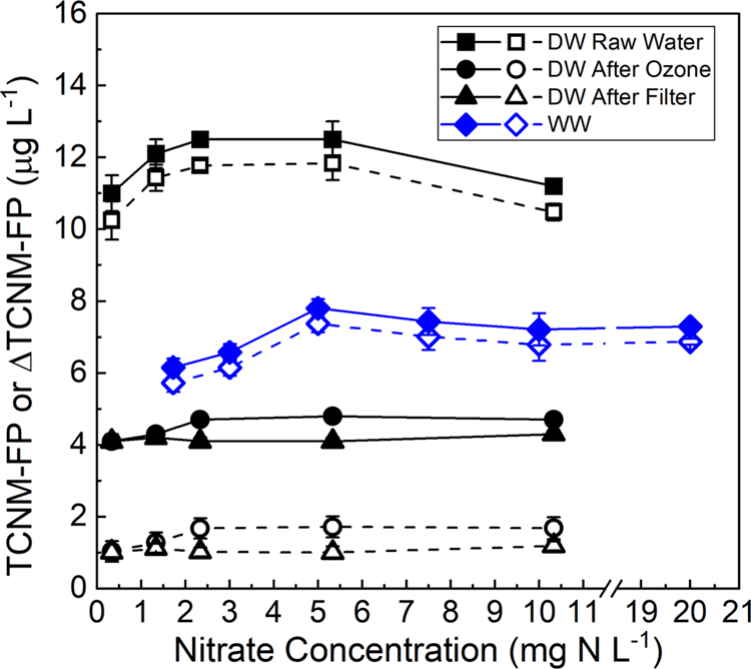
Change of TCNM-FP (solid symbol) and ΔTCNM-FP (open symbol) with respect to nitrate concentrations after irradiation by 100 mJ·cm^–2^ at 222 nm for WW and DW samples. WW sample was first diluted to obtain a low nitrate concentration at 1.7 mg-N·L^–1^ and then spiked with nitrate at different concentrations. DW samples were not diluted and directly spiked with nitrate. ΔTCNM-FP = TCNM-FP_100mJ·cm^–2^_ – TCNM-FP_0mJ·cm^–2^_.

Further experiments were conducted in the real water samples with the adjustment of the nitrate concentration. Unlike the results for model HA and FA, no big increase in TCNM-FP was observed for all four real water samples as the nitrate concentration was increased, after 100 mJ·cm^–2^ irradiation (Figure 3). The change of TCNM-FP was within 27 and 17% for the WW sample and three DW samples, respectively. The increase of TCNM-FP (ΔTCNM-FP) by irradiation was also calculated (Figure 3: open symbols) to account for TCNM-FP of dark control samples. A similar trend was observed because nitrate itself did not affect TCNM-FP. For preozonated samples, TCNM-FP of dark control samples dominated the total TCNM-FP (4–5 μg·L^–1^), and ΔTCNM-FP was in the range of 1–2 μg·L^–1^. Hence, in KrCl* excilamp treatment of water/wastewater, high-level contamination by nitrate may not cause an elevated risk in increasing TCNM-FP, compared with low-level contamination by nitrate. However, these results also indicate that a small amount of nitrate (e.g., around 0.33 mg-N·L^–1^ in this study) already increased TCNM-FP, suggesting that complete elimination of TCNM risk by nitrate is challenging. For other DBPs, combined nitrate and 222 nm irradiation did not significantly change their FP (Figure S15).

For “DW Raw Water” and WW samples, the minimal dependence of TCNM-FP on nitrate concentration compared to HA and FA solutions is potentially caused by two reasons. First, the dramatic increase of the proportion of light absorption attributed to nitrate (*A*_nitrate_, eq S5, Text S7) occurred at a much lower nitrate concentration for DW and WW samples (0–3 mg-N·L^–1^) than for HA and FA solutions (0–10 mg-N·L^–1^) (Figure S16). For example, for *A*_nitrate_ to reach 0.8, it required a nitrate concentration at 2.5 mg-N·L^–1^ for DW and WW samples but 5–6 mg-N·L^–1^ for HA and FA (Figure S16). Hence, the TCNM-FP of HA and FA showed a stronger dependence on the nitrate concentration within the tested range (1–10 mg-N·L^–1^). Second, the concentration of reactive moieties in DW and WW samples for nitration was relatively low compared with those in HA and FA (see details in Section 3.2.3). It should be recognized that nitration in environmental samples is a complex process, which can be affected by the mixture of background water constitutes. Future studies are warranted to investigate the role of each constitute on nitration. For pretreated samples “DW After Ozone” and “DW After Filter”, organics favorable for nitration were limited due to their removal by ozonation, as discussed in Section 3.2.1. Hence, extra nitrate played a minor role in forming more TCNM precursors.

#### 3.2.3 Effects of Real Water Organic Matter

**Figure 4:**
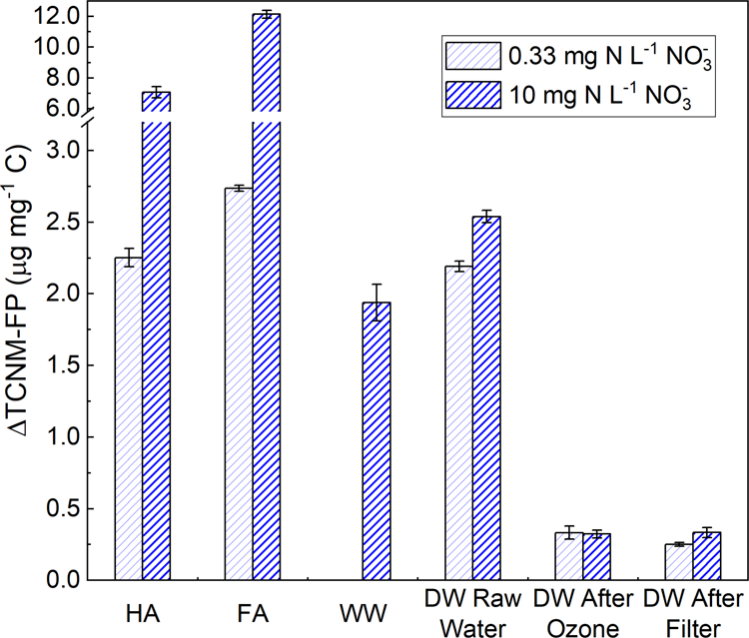
Change of TOC-normalized TCNM-FP after irradiation by 100 mJ·cm^–2^ at 222 nm for HA, FA, WW, and DW samples. Conditions: 3.5 mg-C·L^–1^ TOC and 0.33 or 10 mg-N·L^–1^ nitrate. ΔTCNM-FP = TCNM-FP_100mJ·cm^–2^_ – TCNM-FP_0mJ·cm^–2^_.

To assess the properties of organic matter in WW and DW to be nitrated, further experiments were conducted using samples that were diluted to the same TOC concentration at 3.5 mg-C·L^–1^ and spiked with nitrate at the same concentration (0.33 or 10 mg-N·L^–1^). The TOC concentration was not changed by irradiation even at the highest dose (1000 mJ·cm^–2^) tested in this work for HA and FA (Figure S17), so it was assumed that the TOC remained constant for all samples and that initial TOC was used to calculate the normalized TCNM-FP. Figure 4 shows the increase in the level of TOC-normalized TCNM-FP (μg·mg-C^–1^) by irradiation (100 mJ·cm^–2^) for different samples. At 10 mg-N·L^–1^ nitrate, ΔTCNM-FP followed the order of FA > HA > “DW Raw Water” > WW > “DW After Ozone” > “DW After Filter”. The ΔTCNM-FP values of WW and “DW Raw Water” samples were 3–6 times lower than that for HA and FA, suggesting that organic matter in WW and DW samples was less reactive for nitration. “DW Raw Water” showed higher ΔTCNM-FP than the nitrified WW sample, implying that the nitrified effluent organic matter might feature low nitration ability. The “DW After Ozone” sample featured lower ΔTCNM-FP than its raw water, suggesting that ozonation not only removed TOC but also transformed the organic moieties and decreased their nitration ability. “DW After Filter” showed similar ΔTCNM-FP to “DW After Ozone”, so coagulation/flocculation/filtration did not change the properties of organic matter but only reduced TOC to decrease absolute TCNM-FP (μg·L^–1^) in Figure 2b. It should also be noted that nitrate concentrations affected the conclusion about the ΔTCNM-FP of organic matter. For example, the “DW Raw Water” sample exhibited similar ΔTCNM-FP to HA at 0.33 mg-N·L^–1^ nitrate but lower ΔTCNM-FP at 10 mg-N·L^–1^. Future research should assess the nitration ability of organic matter at each specific nitrate concentration.

To quantitatively evaluate reactive moieties for nitration, SUVA was determined for the real water samples and model NOM. Due to considerable absorption by nitrate and nitrite at 254 nm, their absorption was subtracted in this study for SUVA. As shown in Table S1, both WW and “DW Raw Water” samples featured SUVA values 8–10 times lower than those of HA and FA solutions, suggesting their low aromaticity and potentially low abundance of moieties for nitration. Correlation tests using SUVA and TOC-normalized Δ*T*CNM-FP showed a higher SUVA caused a higher ΔTCNM-FP except HA (Figure S18). Phenolic and aniline-like moieties that are reactive for nitration commonly feature high SUVA. It should be noted that SUVA, a parameter representing the abundance of aromatic moieties, cannot directly indicate the moieties for nitration, because some aromatic compounds (e.g., nitrobenzene) are resistant to nitration.^61^ However, SUVA is still meaningful for controlling nitration moieties and TCNM-FP for utilities. First, low SUVA is a sufficient condition for low nitration ability. In other words, controlling SUVA to a low level will benefit removal of nitration moieties. Second, SUVA is relatively simple to quantify for utilities to monitor this potential risk. Indeed, other analytical methods, such as extraction and analysis of phenolic compounds, should be employed in future studies to systemically evaluate nitration ability of organic matter.^62^

### 3.3 Control of TCNM-FP by the Addition of H_2_O_2_

**Figure 5:**
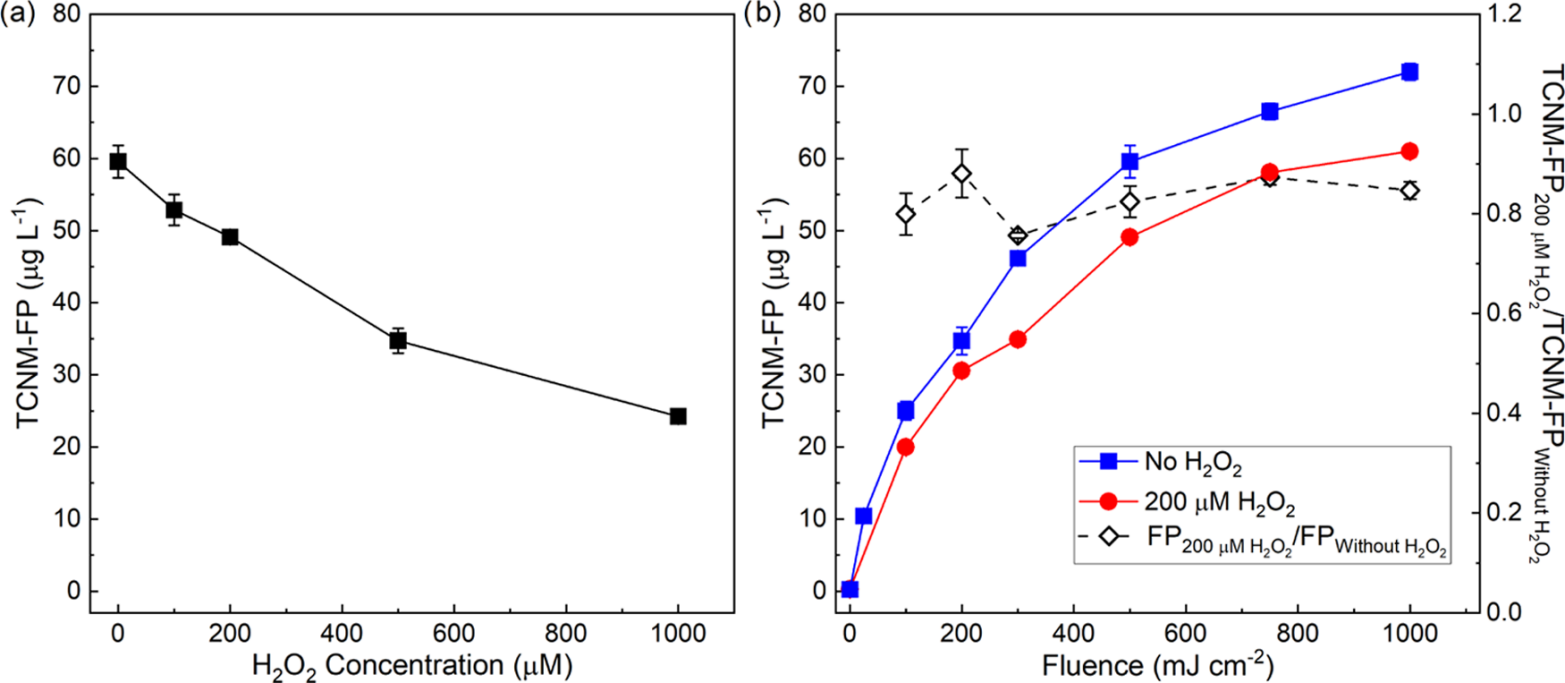
Change of TCNM-FP after irradiation at 222 nm for HA solution with respect to (a) H_2_O_2_ concentration at a fluence of 500 mJ·cm^–2^ or (b) irradiation fluence in the spike of 200 μM H_2_O_2_. Conditions: 3.5 mg-C·L^–1^ TOC, 10 mg-N·L^–1^ nitrate, 100 mg·L^–1^ chloride, 0.1 mg·L^–1^ bromide, and pH 6.8 by 10 mM phosphate. TCNM-FP in the absence of H_2_O_2_ was reproduced from the data in Figure 1b.

To control the risk in forming TCNM precursors by nitrate in 222 nm irradiation, AOP with H_2_O_2_ was tested using HA solutions with varying H_2_O_2_ concentration and irradiation fluence. The selection of UV/H_2_O_2_ is because it is widely used in the United States and Canada to control organic contaminants in drinking water^63^ and wastewater treatment^64^ or reuse.^65,66^ Further, KrCl* excilamps can be utilized with H_2_O_2_ to enable enhanced AOP^20^ and in situ control of TCNM-FP. Figure 5a shows that H_2_O_2_ decreased TCNM-FP in the presence of 10 mg-N·L^–1^ nitrate. A higher H_2_O_2_ concentration resulted in a lower TCNM-FP. At 100–1000 μM (3.4–34 mg·L^–1^) H_2_O_2_, dosages commonly used for AOPs, TCNM-FP was removed by 11–59%. The increase in irradiation fluence did not affect the removal of TCNM-FP (Figure 5b). H_2_O_2_ at 200 μM achieved consistent ∼20% decrease in TCNM-FP for different fluences in the range of 100–1000 mJ·cm^–2^. The addition of H_2_O_2_ may decrease TCNM-FP by (1) light screening by H_2_O_2_ and (2) the decay of nitrating agents, nitration moieties, and/or TCNM precursors by ^•^OH. Light absorption by nitrate was hardly affected by the spike of 100–1000 μM H_2_O_2_ (Table S2). Hence, the control of TCNM-FP mainly resulted from the decay of ^•^NO_2_, organic moieties, and/or TCNM precursors by ^•^OH formed from H_2_O_2_. For water/wastewater treatment, AOP is among the most important processes to control emerging OMPs by reactive radicals.^67,68^ Our results suggested that AOP, such as UV/H_2_O_2_, is also beneficial to controlling the formation of TCNM when KrCl* excilamps are applied. The spike of H_2_O_2_ also decreased FP for other DBPs at high H_2_O_2_ concentrations (500–1000 μM) (Figure S19). However, a low H_2_O_2_ concentration did not greatly change their FP. As for the irradiation dose, an increase of fluence up to 1000 mJ·cm^–2^ did not affect the effect of H_2_O_2_ on decreasing FP for most DBPs, except 1,1-DCP (Figure S20). A dose of 1000 mJ·cm^–2^ at 200 μM H_2_O_2_ removed 40% of 1,1-DCP-FP. As discussed in Section 3.1.2, the oxidation of 1,1-DCP precursors with ^•^OH was potentially the major mechanism.

## 4 Environmental Implications

KrCl* excilamps at 222 nm are promising UV technologies for water treatment with enhanced disinfection, photolysis, and AOP. However, one potential risk associated with KrCl* excilamps is the formation of precursors of nitrogenous disinfection byproducts, such as chloropicrin (TCNM), in the presence of nitrate and/or nitrite. This study revealed that KrCl* excilamps increased the formation potential of TCNM for nitrate- and nitrite-contaminated model NOM solutions (HA or FA) and real water samples, including secondary wastewater effluent, raw drinking water, and partially treated drinking water. At EPA regulation of 10 mg-N·L^–1^ for nitrate, a disinfection dose relevant to water treatment (100 mJ·cm^–2^) at 222 nm increased TCNM formation potential by 15.1–17 times for raw drinking water and secondary wastewater effluent. Mechanistic investigation shows that a similar condition increased the level of TCNM-FP by 7.1–12.1 μg·mg-C^–1^ for HA and FA solutions. Organic properties and nitrate concentrations are two critical factors affecting this risk: FA exhibited a higher increase of TOC-normalized TCNM-FP than HA; 5–10 mg-N·L^–1^ nitrate is the concentration range causing the peak TCNM-FP. Due to low concentrations in the environment, nitrite is projected to exert minor effects. Experiments on drinking water samples in this study suggested that ozonation, coagulation/flocculation/filtration, and addition of H_2_O_2_ are useful tools to decrease TCNM-FP and control this risk with KrCl* excilamps.

Future studies are indeed warranted on three aspects. First, we acknowledge that the results in this study were based on single DW or WW samples; therefore, the conclusions may need modifications in other scenarios with different treatment processes and varied concentrations of background organic matter and constituents. More studies with other types of wastewater (e.g., non-nitrified and denitrified effluents) and drinking water are necessary to fully assess the risk in forming TCNM. Second, understanding the nitration ability of different organic moieties is critical for controlling the formation of TCNM precursors under 222 nm treatment. This study selected two model NOM (HA and FA) and employed SUVA for investigating potent nitration moieties. Larger groups of model organics with different functional groups and other properties, such as the electron-donating capacity and total phenolic content, should be further tested to provide more insights. Lastly, elucidation of the role of different nitrating agents in forming TCNM precursors is needed. The steady-state concentrations of ^•^NO_2_ were estimated in this study for KrCl* excilamps with nitrate. However, other nitrating agents, such as peroxynitrite (ONOO^–^), also existed in nitrate photolysis; hence, their formation at 222 nm and impact on TCNM-FP should be further assessed. Other general issues for KrCl* excilamps caused by nitrate include inhibited disinfection or oxidation due to light screening, short light penetration depth for waters with nitrate pollution, and formation of toxic nitrite. These aspects should be focused on in future work to improve the feasibility of applying KrCl* excilamps to water treatment.

## Supporting Information Available

Chemicals, experimental setup, formation potential test of DBPs, analytical methods, information on real water samples, estimation of steady-state radical concentrations, light absorbance of reactants under experimental conditions, data of TCNM and other DBPs formation potential, TOC concentrations of HA and FA solutions and effect of H_2_O_2_ on formation potential of DBPs (PDF)
