# Supplementary material for: Far-UVC 222 nm Treatment: Effects of Nitrate/Nitrite on Disinfection Byproduct Formation Potential
Source: Environ Sci Technol. 2024 Aug 12;58(34):15311–20. doi: 10.1021/acs.est.4c04258 (PMC11360365; doi:10.1021/acs.est.4c04258)
Supplement: Supplementary file 1 — es4c04258_si_001.pdf [file es4c04258_si_001.pdf]

Supporting Information

Far-UVC 222 nm Treatment: Effects of Nitrate/Nitrite on Disinfection Byproduct Formation Potential

Jiale Xu[1,2,*], Ryan J. Kann[3], Dauda Mohammed[1], and Ching-Hua Huang[2,*]

[1] Department of Civil, Construction and Environmental Engineering, North Dakota State University, Fargo, North Dakota 58102, United States

[2] School of Civil and Environmental Engineering, Georgia Institute of Technology, Atlanta, Georgia 30332, United States

[3] School of Biological Sciences, Georgia Institute of Technology, Atlanta, Georgia 30332, United States

[*]Corresponding authors:

Email: ching-hua.huang@ce.gatech.edu (Ching-Hua Huang);

Email: jiale.xu@ndsu.edu (Jiale Xu)

Number of Pages: 22

Number of Texts: 7

Number of Tables: 3

Number of Figures: 20

Number of References: 7

Text S1. Chemicals

Humic acids and Suwannee River fulvic acids were purchased from MP Biomedicals and International Humic Substances Society, respectively. Sodium phosphate monobasic monohydrate (99.9%), methyl *tert*-butyl ether (MtBE, >99%), sodium nitrate (≥99%), sodium nitrite (≥97%), sodium sulfate (99.0%–100.5%), sodium chloride (99.0%–100.5%), sodium bromide (≥99%), phenol (>99%), nitrobenzene (99%), 4-chlorophenol (99%), 4-chloro-2-nitrophenol (>98.0%), and sodium thiosulfate (≥98%) were supplied from Fisher Chemical. Potassium iodate (99.5%), sodium tetraborate decahydrate (≥99.5%), 2,2'-azino-bis(3-ethylbenzothiazoline-6-sulfonic acid) diammonium salt (≥98%), *tert*-butyl alcohol (≥99%), and horseradish peroxidase were purchased from Sigma-Aldrich. Sodium hypochlorite solution (5%) was purchased from Ricca Chemical. *N*,*N*-Diethyl-*p*-phenylenediamine (DPD) total chlorine reagent powder pillows were obtained from Hach Company. Sodium phosphate dibasic heptahydrate (98–102%) and H_2O_2 solution at 30% w/w were purchased from EMD Millipore. NO_2 gas at 100 ppm in nitrogen gas was obtained from Linde Welding Gas. EPA 501/601 trihalomethanes calibration mix (2000 μg/mL of each THM in methanol) and EPA 551B halogenated volatiles mix (2000 μg/mL of each DBP in acetone) were supplied from Supelco. 1,2-Dibromopropane (>98.0%) was purchased from TCI America. Potassium iodide (99%) was obtained from Thermo Scientific Chemicals. All chemicals were used as received. All aqueous solutions were prepared using ultrapure deionized water.

Text S2. Formation Potential (FP) Test for DBPs

The formation potential (FP) test is a standard method to indicate the amount of DBP precursors in water samples.[1] This study followed a similar but modified procedure developed previously for surveying the nitrogenous DBPs of wastewater samples.[1,2] Sample solutions (20

mL, before and after the KrCl* excilamp irradiation) were buffered at pH 7.2 with 10 mM phosphate buffer and then spiked with NaOCl to achieve a chlorine dose of 10 mg-Cl_2·L^{-1}. The relatively low concentration of chlorine was to prevent the decay of DBPs, especially nitrogenous DBPs, caused by excess chlorine.[3] To account for the excess consumption of chlorine by samples spiked with nitrite, a higher dose of NaOCl (mg-Cl_2·L^{-1}) = 10 + 5 × NO_2^-–N (mg-N·L^{-1}) was used in those experiments. Figure S3 shows that abundant chlorine was provided for the FP tests. For experiments with the spike of H_2O_2, H_2O_2 was quenched by sodium thiosulfate before the FP test. The minimal concentration of H_2O_2 was confirmed by a colorimetric method using 2,2′-azino-bis(3-ethylbenzothiazoline)-6-sulfonate (ABTS) and horseradish peroxidase as described previously.[4]

Each FP test lasted for 24 h and residual chlorine was quenched by sodium thiosulfate. Ten disinfection byproducts (DBPs), chloropicrin (TCNM), four trihalomethanes (THMs), including chloroform (TCM), dichlorobromomethane (DCBM), dibromochloromethane (DBCM), and bromoform (TBM), three haloacetonitriles (HANs), including dichloroacetonitrile (DCAN), bromochloroacetonitrile (BCAN), and dibromoacetonitrile (DBAN), and two haloketones (HKs), including 1,1-dichloropropanone (1,1-DCP) and 1,1,1-trichloropropanone (1,1,1-TCP), were analyzed for all samples. Briefly, DBPs in 20 mL of samples were extracted with 2 mL of methyl *tert*-butyl ether (MtBE) with the spike of the internal standard 1,2-dibromopropane and 10 g sodium sulfate.[2] The MtBE extracts were analyzed by a gas chromatography-electron capture detector (HP 6890 GC-ECD) with a DB-5MS column. The detailed method was described in our previous study.[2] The quantification detection limit was 0.1 μg·L^{-1} for each DBP.

Text S3. Analytical Methods

TOC concentrations of HA, FA, WW samples, and DW samples were measured using a Shimadzu TOC analyzer (TOC-ASI-L; Kyoto, Japan). Nitrate and nitrite concentrations in samples were determined by the Hach cadmium reduction method (Method 8039) and the Hach diazotization method (Method 10019), respectively. Specific UV absorbance (SUVA) was determined by taking the ratio between the absorbance at 254 nm and the TOC concentration. Chlorine concentration at the end of FP tests was measured using Hach *N*,*N*-diethyl-*p*-phenylenediamine (DPD) total chlorine method. The concentrations of phenol, nitrobenzene, 4-chlorophenol, and 4-chloro-2-nitrophenol were measured by an Agilent 1100 high-performance liquid chromatography (HPLC)/diode-array detector (DAD) system equipped with an Agilent Zorbax SB-C18 column (2.1 × 150 mm, 5 μm) using an isocratic mobile phase: 25% of acetonitrile and 75% 0.1% (v/v) formic acid in water at a flow rate of 0.3 $mL{\cdot}min^{-1}$.

Text S4. Estimation of Steady-State Concentrations of $^{\bullet}OH$ and $^{\bullet}NO_2$ and the Contribution of Other Reactive Nitrogen Species

To determine the concentrations of $^{\bullet}OH$ and $^{\bullet}NO_2$, and the role of other reactive nitrogen species (RNS), probe compounds nitrobenzene (NB), 4-chlorophenol (4-CP), and phenol (PHE) were spiked into HA or FA solutions at 1.0 μM. These solutions contained 3.5 $mg\text{-}C{\cdot}L^{-1}$ TOC, 100 $mg{\cdot}L^{-1}$ chloride, 0.1 $mg{\cdot}L^{-1}$ bromide, and 5 or 40 $mg\text{-}N{\cdot}L^{-1}$ nitrate, and buffered at pH 6.8 by 10 mM phosphate. The solutions were spiked with one of the prob compounds and exposed to 222-nm irradiation and the concentrations of probe compound were monitored over time. NB is highly reactive with $^{\bullet}OH$ ($k = 3.9 \times 10^9$ $M^{-1}{\cdot}s^{-1}$) but inert with RNS, and hence it can be used to estimate the concentrations of $^{\bullet}OH$. 4-CP can react with both $^{\bullet}OH$ ($k = 7.6 \times 10^9$ $M^{-1}{\cdot}s^{-1}$) and $^{\bullet}NO_2$ ($k = 1.1 \times 10^4$ $M^{-1}{\cdot}s^{-1}$), so combining the decays of NB and 4-CP can be used to obtain the concentration

of $^{\bullet}NO_2$. Lastly, PHE is reactive to $^{\bullet}OH$ ($k = 6.6 \times 10^9$ $M^{-1} \cdot s^{-1}$), $^{\bullet}NO_2$ ($k = 3.2 \times 10^3$ $M^{-1} \cdot s^{-1}$), and other RNS (e.g., $ONOO^-/HOONO$), which was used to assess the decay rate by other RNS. The steady-state concentrations of $^{\bullet}OH$ and $^{\bullet}NO_2$ can be calculated by the following equations:

$$k_{Probe} = k_{photolysis} + k_{Probe,\,OH}C_{OH} + k_{Probe,\,NO2}C_{NO2} + k_{Probe,\,RNS} \quad \text{Equation S1}$$

$$k_{photolysis} = \frac{E_{avg}}{C_{Probe}d}\Phi_{222}\left[1\text{-}10^{-abs \times d}\right]\left(\frac{\varepsilon_{Probe}C_{Probe}}{abs \times d}\right) \quad \text{Equation S2}$$

$$abs = \left(\sum \varepsilon_{Probe}C_{Probe}\right) + \varepsilon_{Nitrate}C_{Nitrate} + a_{\lambda} \quad \text{Equation S3}$$

where k_{Probe} (s^{-1}) is observed pseudo-first-order decay rate constant for NB, 4-CP, or PHE; $k_{photolysis}$ (s^{-1}) is the direct photolysis rate; $k_{Probe,\,OH}$ and $k_{Probe,\,NO2}$ (M^{-1} s^{-1}) are the second-order rate constants for probe with $^{\bullet}OH$ and $^{\bullet}NO_2$, respectively; $k_{Probe,\,RNS}$ (s^{-1}) is the first-order decay rate constant for probe with other RNS; C_{OH}, C_{NO2}, $C_{Nitrate}$, and C_{Probe} (M) are concentrations for $^{\bullet}OH$, $^{\bullet}NO_2$, nitrate, and probe compound, respectively; E_{avg} (Einstein dm^{-2} s^{-1}) is the averaged fluence rate of 3.15×10^{-7} Einstein$\cdot m^{-2} \cdot s^{-1}$ at 222 nm; d (dm) is effective path length of 1.8 cm; Φ_{222} (dimensionless) is the quantum yield for probe compound at 222 nm; ε_{Probe} and $\varepsilon_{Nitrate}$ ($M^{-1} \cdot cm^{-1}$) is the molar absorption coefficient of probe compound and nitrate at 222 nm, respectively; abs (cm^{-1}) is the total light absorption per cm for the solution; and a_{λ} (cm^{-1}) is the light absorption per cm by humic acids (HA) or fulvic acids (FA) at 3.5 mg-C$\cdot L^{-1}$. By applying each probe to equations S1, S2, and S3 for all three probes, the steady-state concentrations of $^{\bullet}OH$ (C_{OH}) and $^{\bullet}NO_2$ (C_{NO2}) as well as the decay rate constant for phenol by other RNS ($k_{PHE,\,RNS}$) were obtained for HA and FA solutions at 5 or 40 mg-N$\cdot L^{-1}$ nitrate. The values of parameters are shown in Table S3.

Text S5. Purging Experiments to Assess the Effects of Nitrate on the Steady-State Concentration of $^{\bullet}NO_2$

To assess whether nitrate reacts with $^{\bullet}NO_2$, NO_2 gas at 100 ppm was purged into 10 μM 4-chlorophenol solutions with the spike of 0– 40 mg-N·L^{-1} nitrate and buffered at pH 6.8 by 10 mM phosphate in deionized water. The gas flow rate was 100 mL·min^{-1}, and the solution volume was 100 mL. The formation rates of 4-chloro-2-nitrophenol and nitrite can indicate the level of steady-state concentration of $^{\bullet}NO_2$ in the solution. Results are shown in Figure S6.

Text S6. Quenching Experiments

To evaluate the effects of $^{\bullet}OH$ on the nitration process, *tert*-butyl alcohol (TBA) as an efficient quencher of $^{\bullet}OH$ was employed. Briefly, 5 mM TBA was spiked before irradiation into HA (3.5 mg-C·L^{-1}) solutions buffered at pH 6.8 by 10 mM phosphate and spiked with 100 mg·L^{-1} chloride and 0.1 mg·L^{-1} bromide. Nitrate concentration was 5 or 40 mg-N·L^{-1}. After irradiation by 100 mJ·cm^{-2}, FP test was conducted to measure TCNM-FP. For the control experiment, TBA was spiked after irradiation and before FP test to ensure the same TOC concentration during FP test. Results are shown in Figure S7. To evaluate the effect of TBA on radical concentrations, further experiments were conducted using probe compounds as described in Text S4 to estimate the change of the concentrations of $^{\bullet}OH$ and $^{\bullet}NO_2$ as well as the role of other reactive nitrogen species (RNS) by TBA. Results are shown in Figure S5.

Text S7. Estimation of Light Absorbance by the Entire Solution, Organic Matter, Nitrate, and Hydrogen Peroxide

The light absorption by the entire solution (A_{Total}, dimensionless), organic matter (A_{OM}), nitrate ($A_{Nitrate}$), and hydrogen peroxide ($A_{Peroxide}$) was estimated using the following equations[5]:

$$A_{Total} = 1\text{-}10^{-(\varepsilon_{Nitrate}C_{Nitrate} + \varepsilon_{Peroxide}C_{Peroxide} + a_{\lambda})d} \qquad \text{Equation S4}$$

$$A_{Nitrate} = \left[1\text{-}10^{-(\varepsilon_{Nitrate}C_{Nitrate} + \varepsilon_{Peroxide}C_{Peroxide} + a_{\lambda})d}\right]\left(\frac{\varepsilon_{Nitrate}C_{Nitrate}}{\varepsilon_{Nitrate}C_{Nitrate} + \varepsilon_{Peroxide}C_{Peroxide} + a_{\lambda}}\right) \quad \text{Equation S5}$$

$$A_{Peroxide} = \left[1\text{-}10^{-(\varepsilon_{Nitrate}C_{Nitrate} + \varepsilon_{Peroxide}C_{Peroxide} + a_{\lambda})d}\right]\left(\frac{\varepsilon_{Peroxide}C_{Peroxide}}{\varepsilon_{Nitrate}C_{Nitrate} + \varepsilon_{Peroxide}C_{Peroxide} + a_{\lambda}}\right) \quad \text{Equation S6}$$

$$A_{OM} = \left[1\text{-}10^{-(\varepsilon_{Nitrate}C_{Nitrate} + \varepsilon_{Peroxide}C_{Peroxide} + a_{\lambda})d}\right]\left(\frac{a_{\lambda}}{\varepsilon_{Nitrate}C_{Nitrate} + \varepsilon_{Peroxide}C_{Peroxide} + a_{\lambda}}\right) \quad \text{Equation S7}$$

where $C_{Nitrate}$ and $C_{Peroxide}$ (M) are concentrations of nitrate and hydrogen peroxide, respectively; $\varepsilon_{Nitrate}$ and $\varepsilon_{Peroxide}$ ($M^{-1}\cdot cm^{-1}$) are molar absorption coefficient of nitrate (2747 $M^{-1}\cdot cm^{-1}$) and hydrogen peroxide (99 M^{-1} cm^{-1}) at 222 nm[6], respectively; d (cm) is effective path length of 1.8 cm; and a_{λ} (cm^{-1}) is the absorbance by background water matrix at 222 nm. The a_{λ} value is shown in Table S1. For HA and FA, a concentration of 3.5 mg-C·L^{-1} TOC was used. For WW, the calculation was based on sample after dilution to 3.5 mg-C·L^{-1} TOC. For three DW samples, original samples were used.

Table S1. Water quality data of wastewater and drinking water samples in this study.

Samples	Nitrate[a] (mg-N·L^{-1})	Nitrite[a] (mg-N·L^{-1})	TOC[a] (mg-C·L^{-1})	SUVA[b] (L·mg-C^{-1}·m^{-1})	Absorbance by background water matrix at 222 nm excluding nitrate and nitrite[c], a_λ (cm^{-1})
Humic acid	–	–	–	7.395	0.296
Suwannee River fulvic acid	–	–	–	4.428	0.235
WW	15.76	0.04	32.2	0.460	0.072
DW Raw Water	0.33	<0.01	4.9	0.561	0.067
DW After Ozone	0.33	<0.01	4.2	0.217	0.042
DW After Filter	0.33	<0.01	3.8	0.159	0.035

[a] The detection limit is 0.01 mg-N·L^{-1}, 0.01 mg-N·L^{-1}, and 0.2 mg-C·L^{-1} for nitrate, nitrite, and TOC, respectively.

[b] Absorption by nitrate and nitrite was excluded from SUVA.

[c] For HA and FA, a_λ was calculated as the absorbance at 3.5 mg-C·L^{-1}. For WW, the sample was diluted to 3.5 mg-C·L^{-1}. For the other DW samples, the sample was directly used. Then, the absorbance was measured at 222 nm. The value of a_λ was calculated by subtracting the calculated light absorption by nitrate and nitrite at original concentrations.

Table S2. Light absorption by nitrate, hydrogen peroxide, and humic acid in the spike of hydrogen peroxide. Calculation is described in Text S7.

H_2O_2 concentration (μM)	A_{OM} (–)	$A_{Nitrate}$ (–)	$A_{Peroxide}$ (–)
0	0.131	0.869	0.000
100	0.130	0.865	0.004
200	0.130	0.861	0.009
500	0.128	0.850	0.021
1000	0.126	0.832	0.042

Table S3. Parameters for determining steady-state concentrations of $^{\bullet}OH$ and $^{\bullet}NO_2$ as well as the decay rate constant for phenol by other RNS.[2, 6, 7]

Compounds	ε (M^{-1}·cm^{-1})	Φ_{222}	a_λ (cm^{-1})	$k_{Probe, OH}$ (M^{-1}·s^{-1})	$k_{Probe, NO2}$ (M^{-1}·s^{-1})	$k_{Probe, RNS}$ (s^{-1})
NB	2288	0.0364	–	3.9×10^9	Negligible	Negligible
4-CP	9945	0.6579	–	7.6×10^9	1.1×10^4	Negligible
PHE	2957	0.0724	–	6.6×10^9	3.2×10^3	To be calculated
HA	–	–	0.296[a]	–	–	–
FA	–	–	0.235[a]	–	–	–
Nitrate	2747	–	–	–	–	–

[a] Data was reproduced from Table S1.

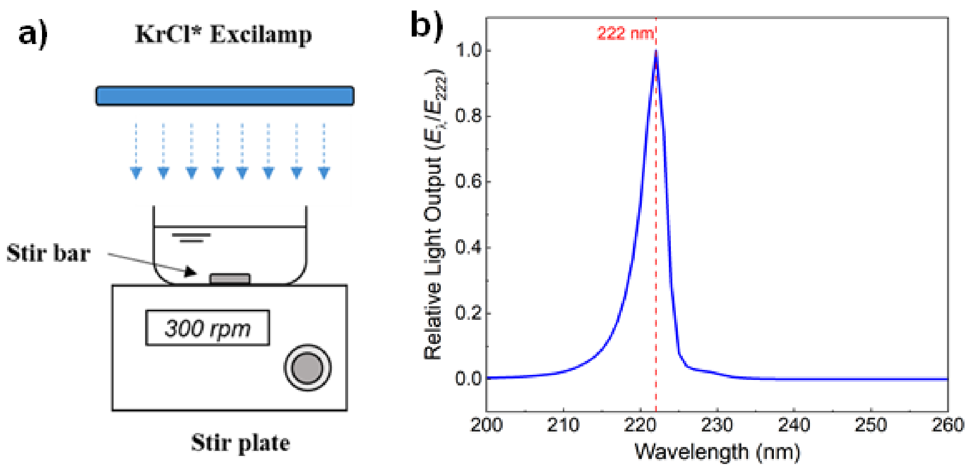

Figure S1. (a) Bench-scale UV setup with KrCl* excilamp and (b) spectrum of emitted light from KrCl* excilamp. E_λ (Einstein m^{-2} s^{-1}) is the incident fluence rate at λ nm, and E_{222} (Einstein·m^{-2}·s^{-1}) is the incident fluence rate at 222 nm.

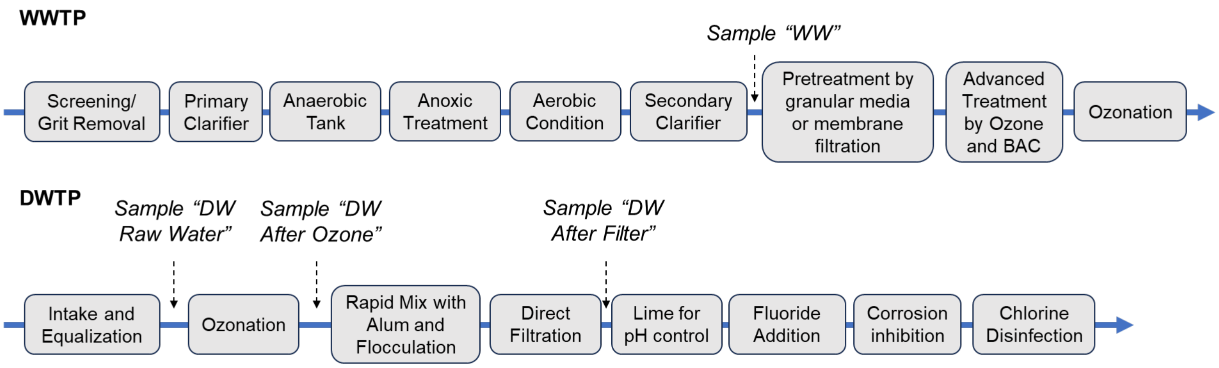

Figure S2. Treatment processes for the WWTP and the DWTP sampled in this study and the sampling locations.

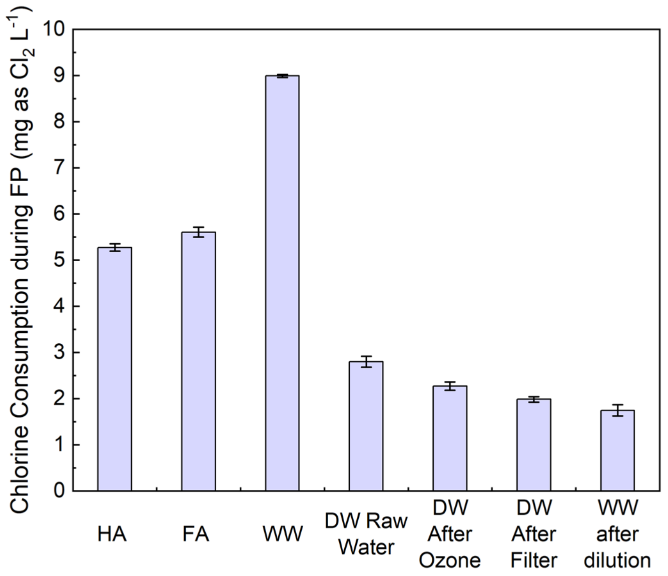

Figure S3. Chlorine consumption after formation potential test (24 h) by model NOM solutions and real water samples after irradiation by 100 mJ·cm^{-2}. HA and FA solutions: 3.5 mg-C·L^{-1} TOC, 10 mg-N·L^{-1} nitrate, 100 mg·L^{-1} chloride, 0.1 mg·L^{-1} bromide, and pH 6.8 by 10 mM phosphate. WW, DW Raw Water, DW After Ozone, and DW After Filter were original samples. WW after dilution was the WW sample diluted to reach 3.5 mg-C·L^{-1} and 1.7 mg-N·L^{-1} nitrate.

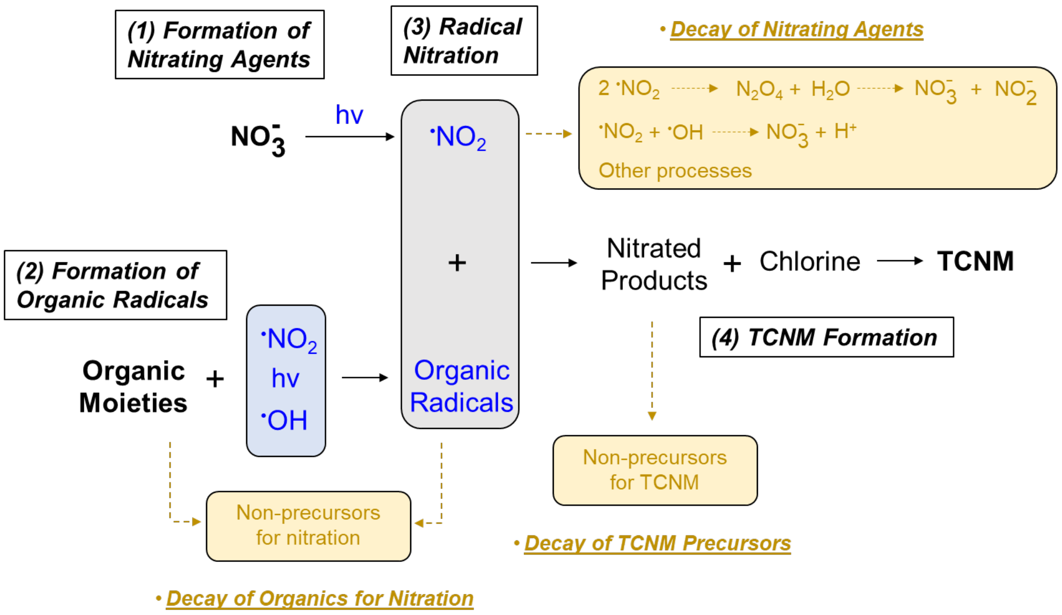

Figure S4. Steps for the formation of TCNM precursors and TCNM in combined UV and chlorination processes, as well as processes for the decay of nitrating agents, organics for nitration, and TCNM precursors in the system.

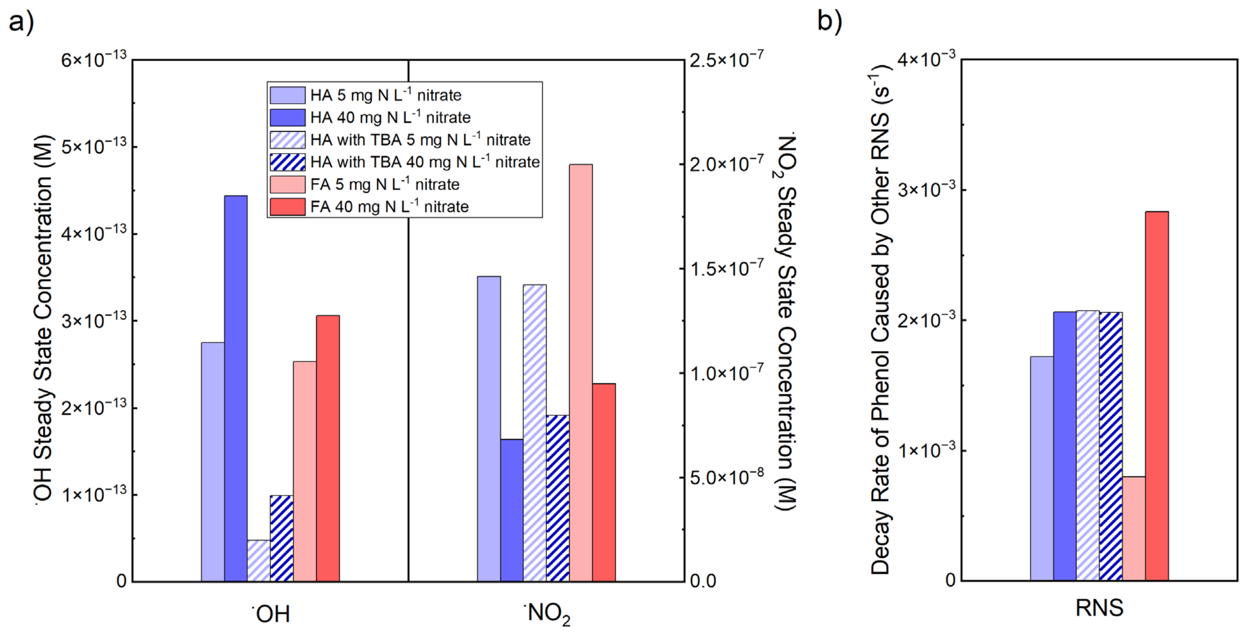

Figure S5. The steady-state concentrations of $^{\bullet}$OH and $^{\bullet}NO_2$ (a), as well as the decay rate of phenol (b) caused by other reactive nitrogen species in the presence or absence of 5 mM *tert*-butyl alcohol (TBA). Conditions: HA or FA solution at 3.5 mg-C·L^{-1} TOC, 100 mg·L^{-1} chloride, 0.1 mg·L^{-1} bromide, and pH 6.8 by 10 mM phosphate. Phenol, 4-chlorophenol, and nitrobenzene were spiked together at 1.0 μM.

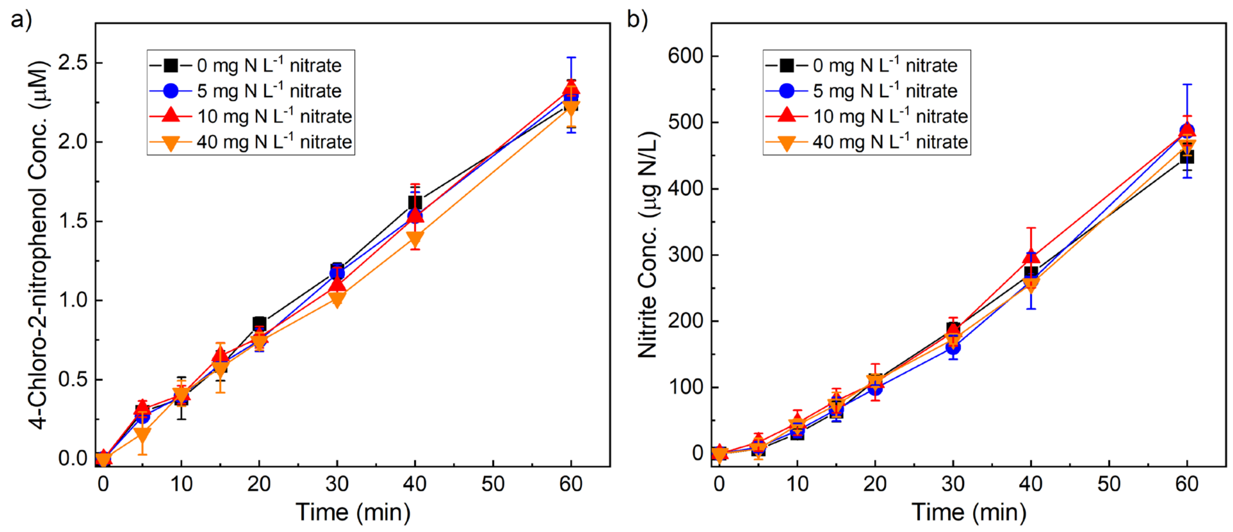

Figure S6. Formation of (a) 4-chloro-2-nitrophenol and (b) nitrite from purging experiments at different nitrate concentrations from 10 μM 4-chlorophenol solutions buffered at pH 6.8 by 10 mM phosphate. Conditions: 100 ppm NO_2 gas in nitrogen gas and 100 mL·min^{-1} flow rate.

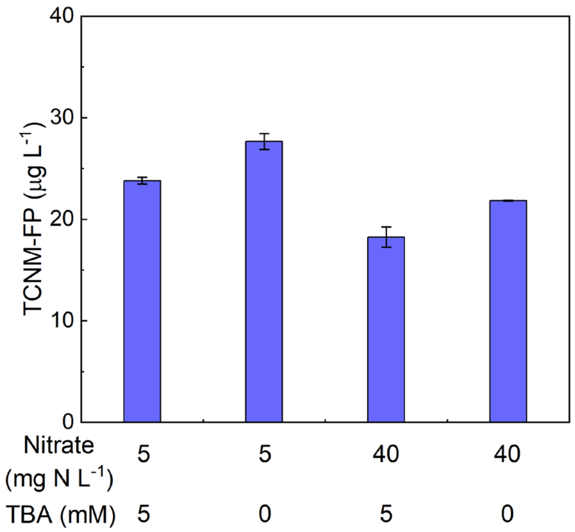

Figure S7. TCNM-FP for HA solutions (3.5 mg-C·L^{-1}) after 100 mJ·cm^{-2} irradiation at 222 nm with the spike of 5 mM TBA before irradiation. For the control experiment (TBA 0 mM in the figure), TBA was spiked after irradiation and before FP test to ensure the same TOC concentration during FP test. Conditions: 3.5 mg-C·L^{-1} HA at pH 6.8 buffered by 10 mM phosphate, 5 or 40 mg-N·L^{-1} nitrate, 100 mg·L^{-1} chloride, and 0.1 mg·L^{-1} bromide.

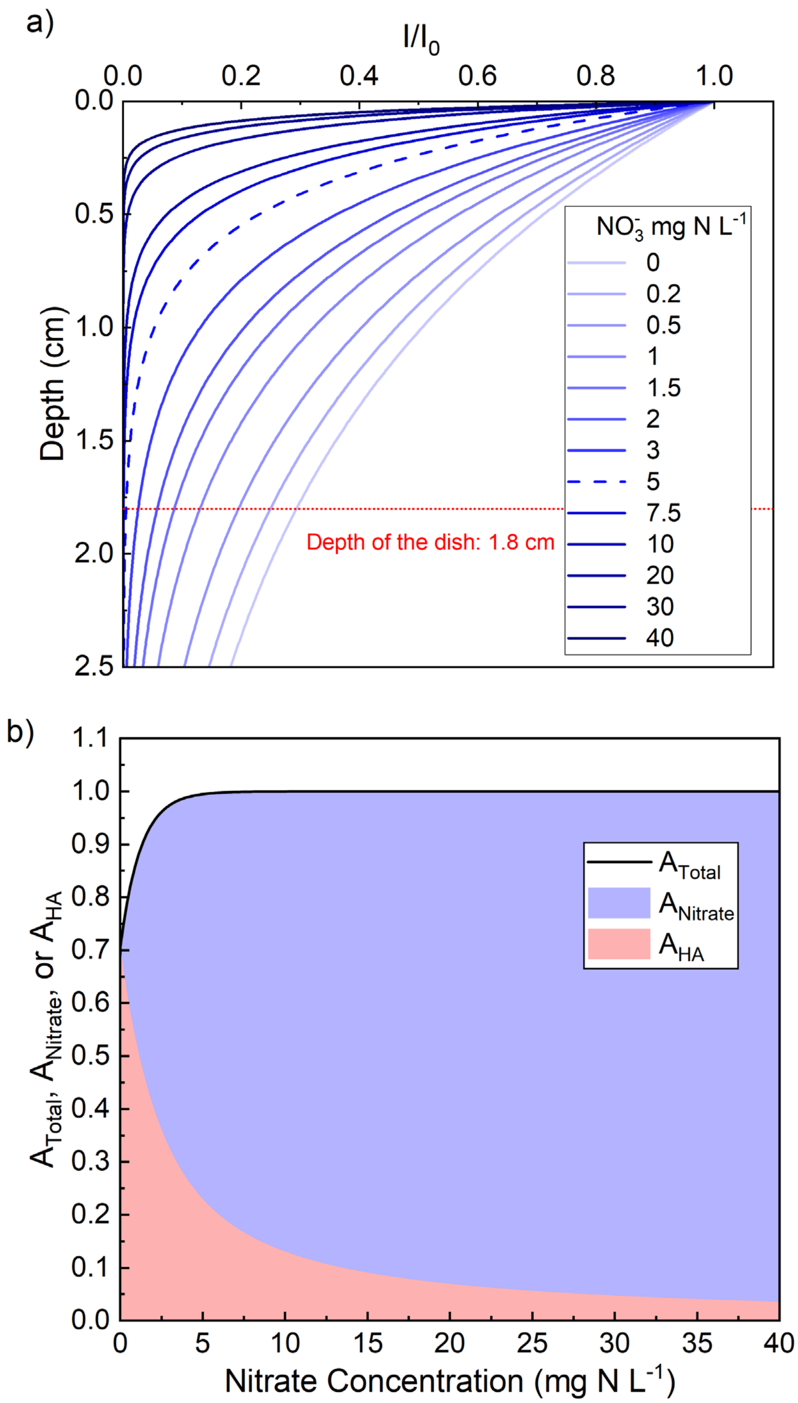

Figure S8. (a) The ratio of residue light intensity (I) to the total light intensity at solution surface (I_0) and (b) the absorption of light by the solution, nitrate, and HA in the system (by 1.8 cm solution) at varying nitrate concentrations for HA solutions at 222 nm. Conditions: 3.5 mg-C·L^{-1} TOC, 100 mg·L^{-1} chloride, 0.1 mg·L^{-1} bromide, and pH 6.8 by 10 mM phosphate. Calculation is described in Text S7, but the term for hydrogen peroxide was not considered.

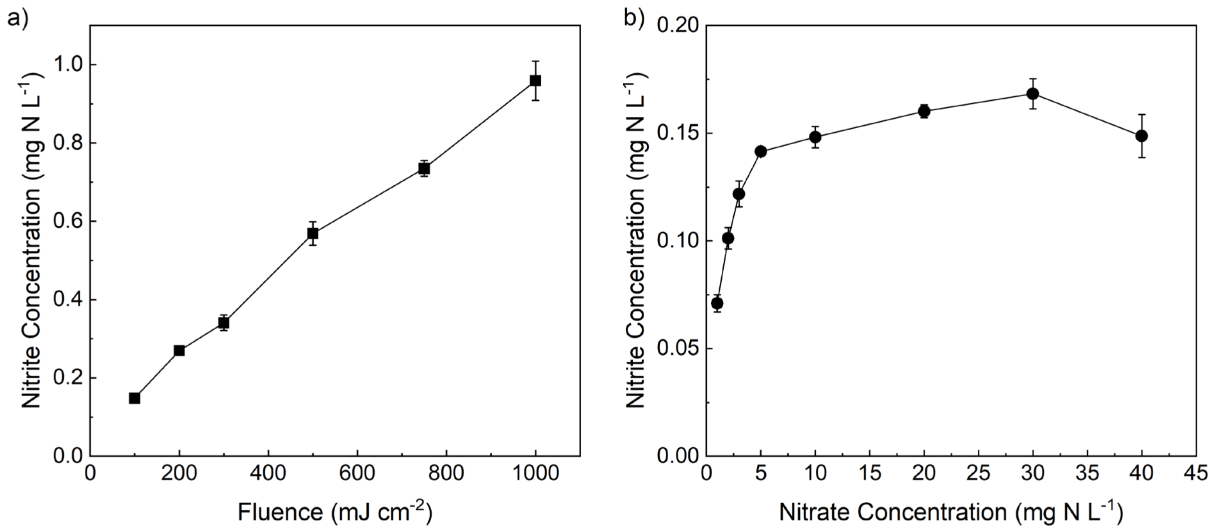

Figure S9. Nitrite concentrations after irradiation (a) at varying fluences and (b) at varying nitrate concentrations for HA solution. Conditions: 3.5 mg-C·L^{-1} TOC, 10 mg-N·L^{-1} nitrate, 100 mg·L^{-1} chloride, 0.1 mg·L^{-1} bromide, and pH 6.8 by 10 mM phosphate; (a) 10 mg-N·L^{-1} nitrate and (b) 100 mJ·cm^{-2} fluence.

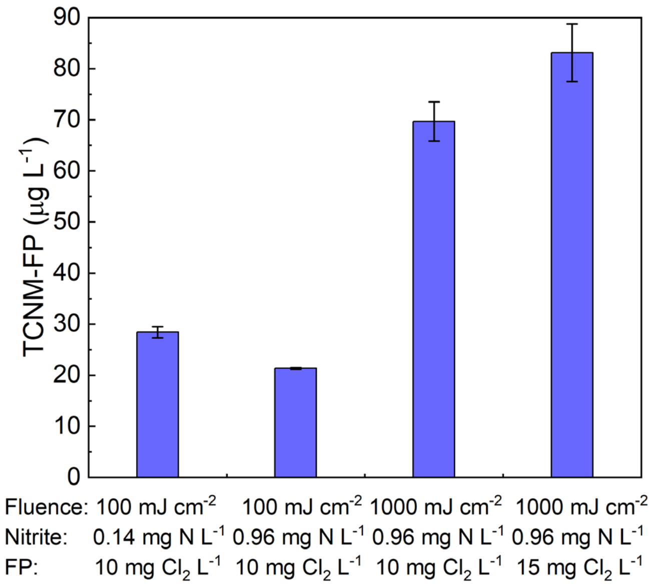

Figure S10. TCNM-FP for HA solutions (3.5 mg-C·L^{-1}) after 100 and 1000 mJ·cm^{-2} irradiation at 222 nm. One sample after 100 mJ·cm^{-2} irradiation was spiked with the same nitrite concentration to that after 1000 mJ·cm^{-2}. For one sample after 1000 mJ·cm^{-2} irradiation, a high chlorine does was used to account for the high nitrite concentration. Conditions: 3.5 mg-C·L^{-1} HA at pH 6.8 buffered by 10 mM phosphate, 100 mg·L^{-1} chloride, and 0.1 mg·L^{-1} bromide.

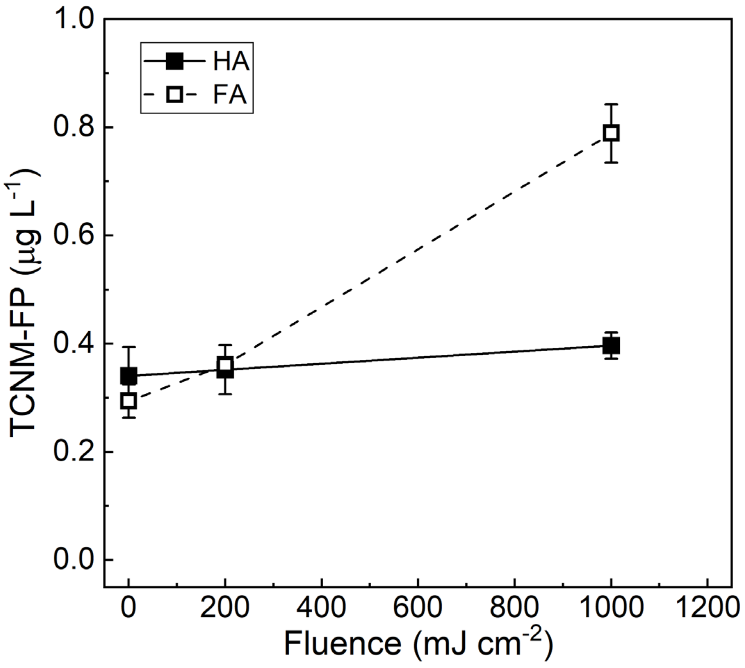

Figure S11. TCNM-FP of HA and FA solutions in the absence of nitrate and nitrite with respect to varying irradiation fluence. Conditions: 3.5 mg-C·L^{-1} TOC, 100 mg·L^{-1} chloride, 0.1 mg·L^{-1} bromide, and pH 6.8 by 10 mM phosphate.

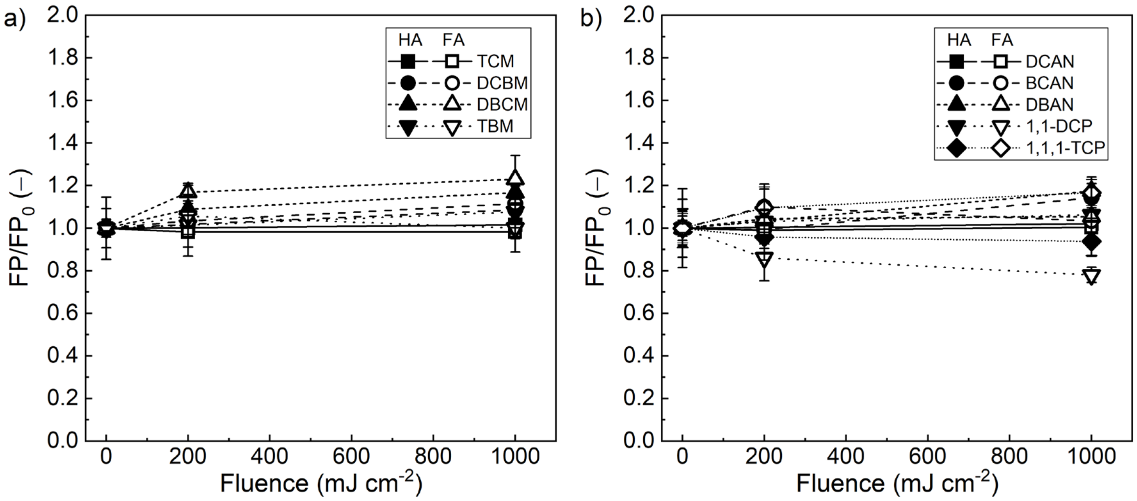

Figure S12. FP for THMs, HANs and HKs of HA and FA solutions in the absence of nitrate and nitrite with respect to varying irradiation fluence. Conditions: 3.5 mg-C·L^{-1} TOC, 100 mg·L^{-1} chloride, 0.1 mg·L^{-1} bromide, and pH 6.8 by 10 mM phosphate.

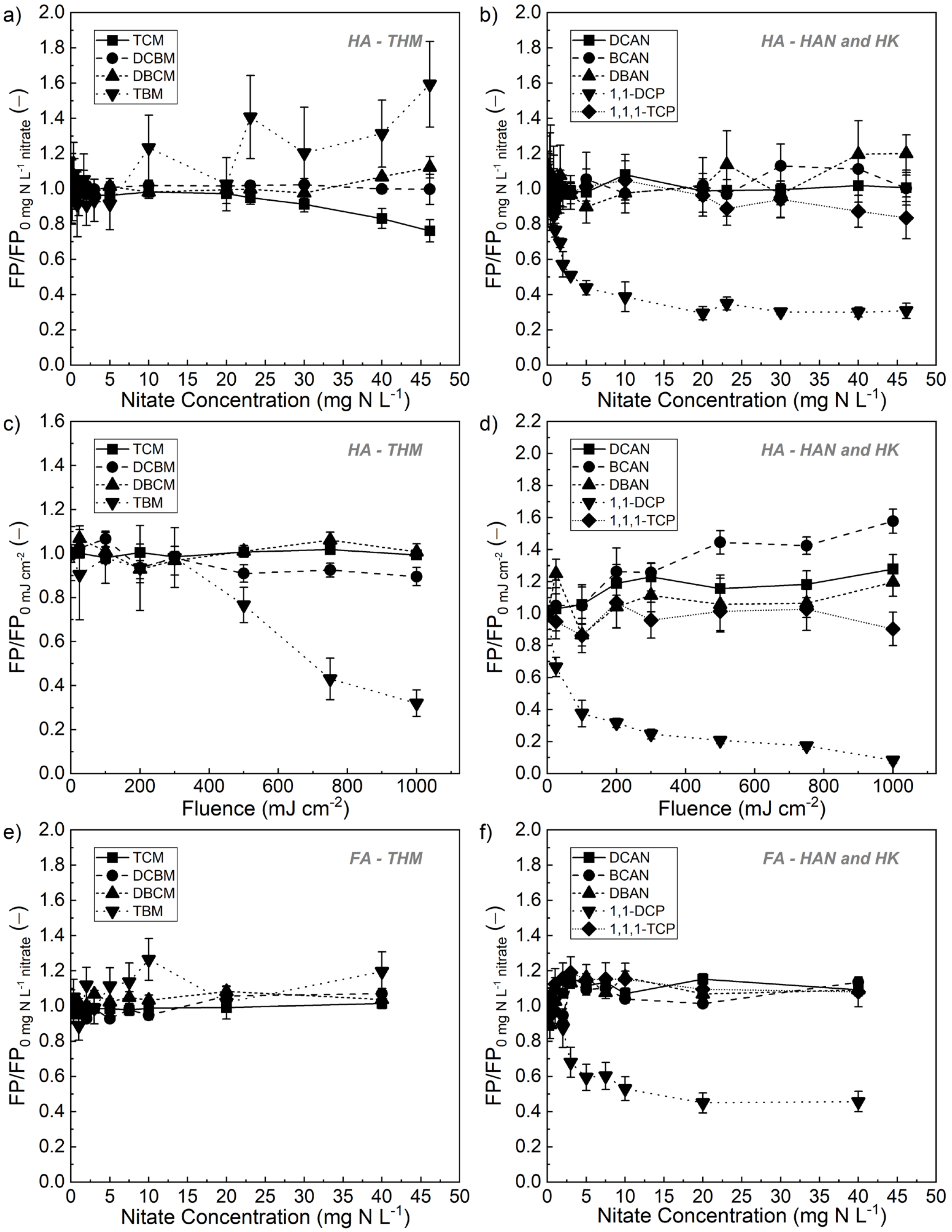

Figure S13. Change of FP for THMs, HANs and HKs for HA and FA solutions at varying nitrate concentrations or varying irradiation fluences. Conditions: 3.5 mg-C·L^{-1} TOC, 100 mg·L^{-1} chloride, 0.1 mg·L^{-1} bromide, and pH 6.8 by 10 mM phosphate. (a)-(b) and (e)-(f): fluence was 100 mJ·cm^{-2}. (c)-(d): 10 mg-N·L^{-1} nitrate.

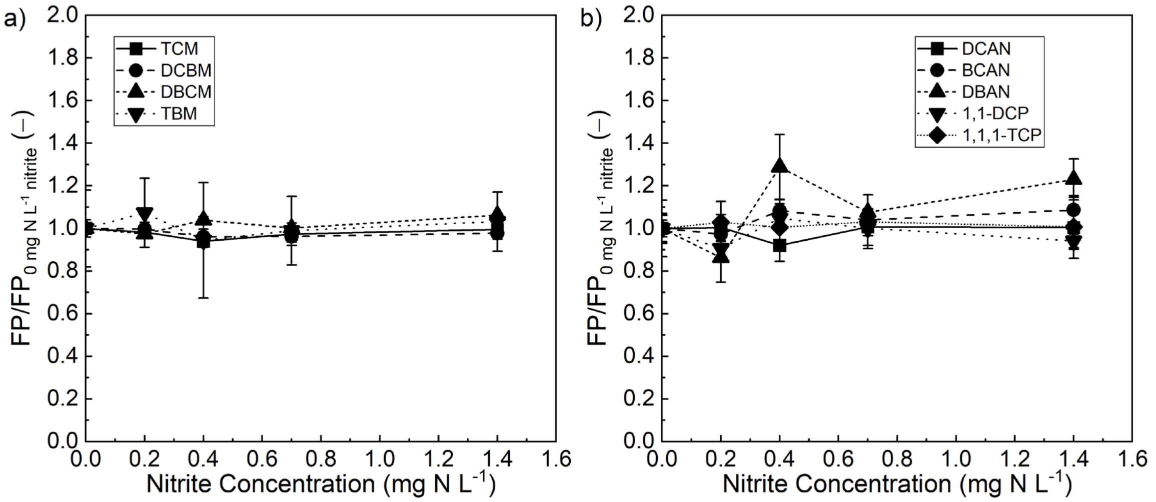

Figure S14. Change of FP for THM, HAN and HK for HA solutions at varying nitrite concentrations at a fluence of 100 mJ·cm^{-2}. Conditions: 3.5 mg-C·L^{-1} TOC, 100 mg·L^{-1} chloride, 0.1 mg·L^{-1} bromide, and pH 6.8 by 10 mM phosphate.

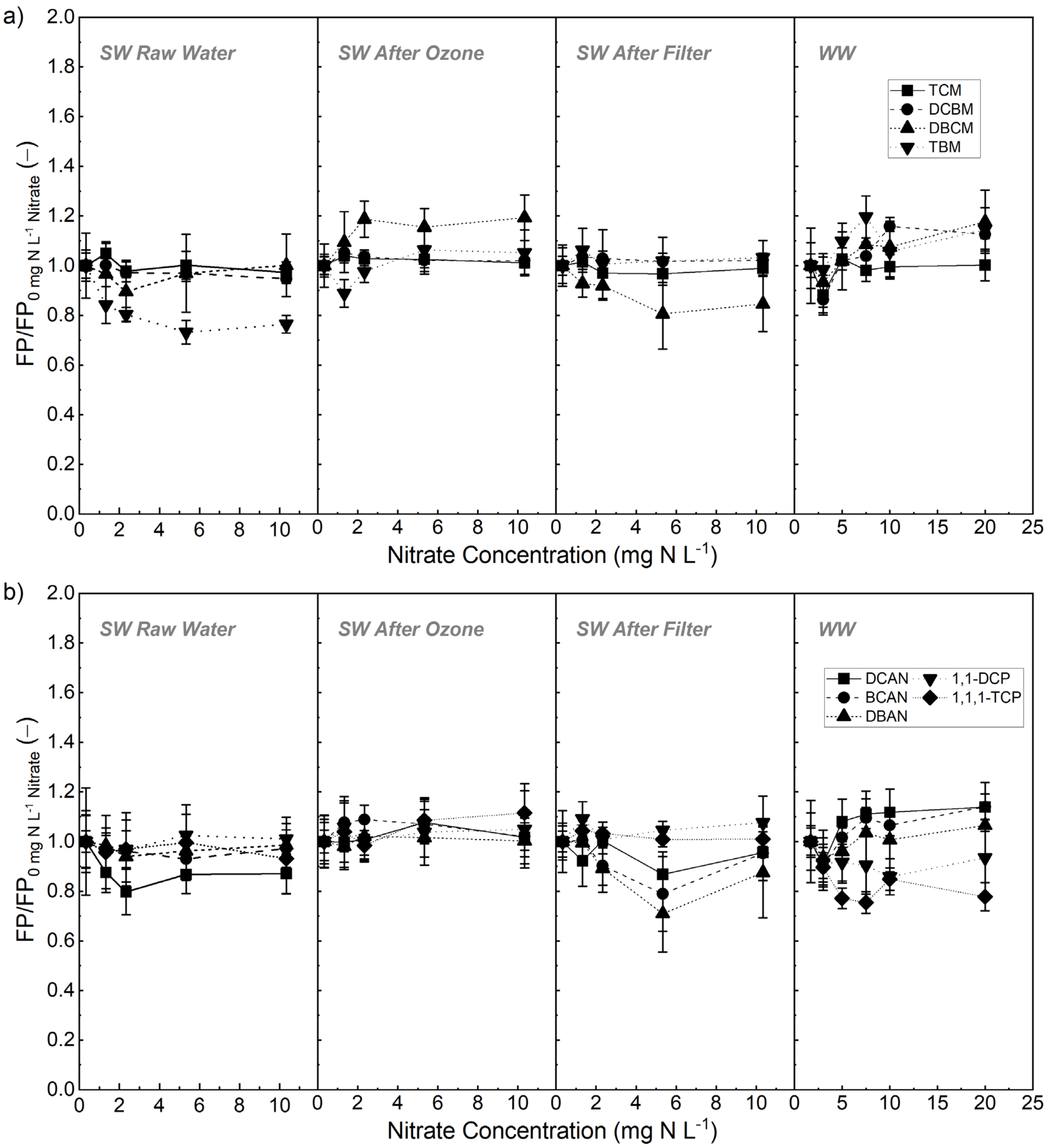

Figure S15. Change of FP for THM, HAN and HK for WW and DW samples at varying nitrate concentrations at a fluence of 100 mJ·cm^{-2}. WW sample was firstly diluted to obtain a low nitrate concentration at 1.7 mg-N·L^{-1} and then spiked with nitrate at different concentrations. DW samples were not diluted and directly spiked with nitrate.

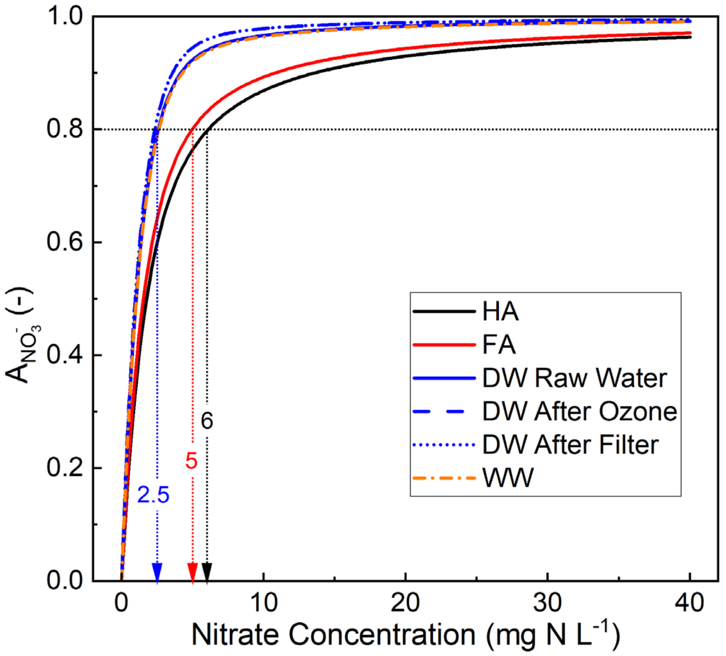

Figure S16. Calculated absorption of light at 222 nm by nitrate in different solutions by the depth of 1.8 cm. HA and FA solutions: 3.5 mg-C·L^{-1} TOC and pH 6.8 by 10 mM phosphate. For WW, the calculation was based on samples after dilution to 3.5 mg-C·L^{-1} TOC. For three DW samples, the calculation was for the original samples without dilution. The calculation was conducted using equation S5 without the term for hydrogen peroxide.

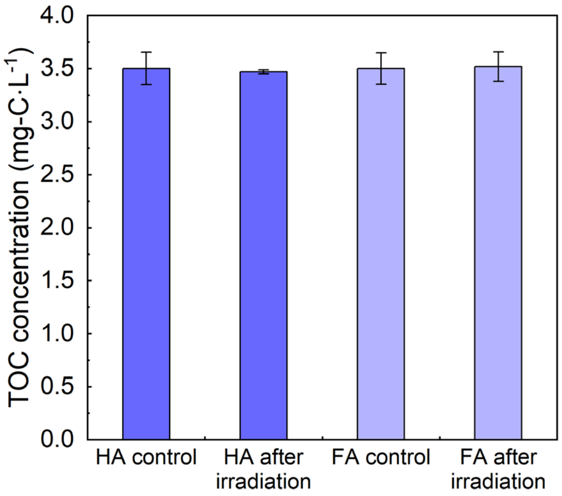

Figure S17. Chang in TOC concentrations of HA and FA solutions after irradiation by 1000 mJ·cm^{-2}. Conditions: HA or FA solution at 3.5 mg-C·L^{-1} TOC, 100 mg·L^{-1} chloride, 0.1 mg·L^{-1} bromide, 10 mg-N·L^{-1} nitrate, and pH 6.8 by 10 mM phosphate.

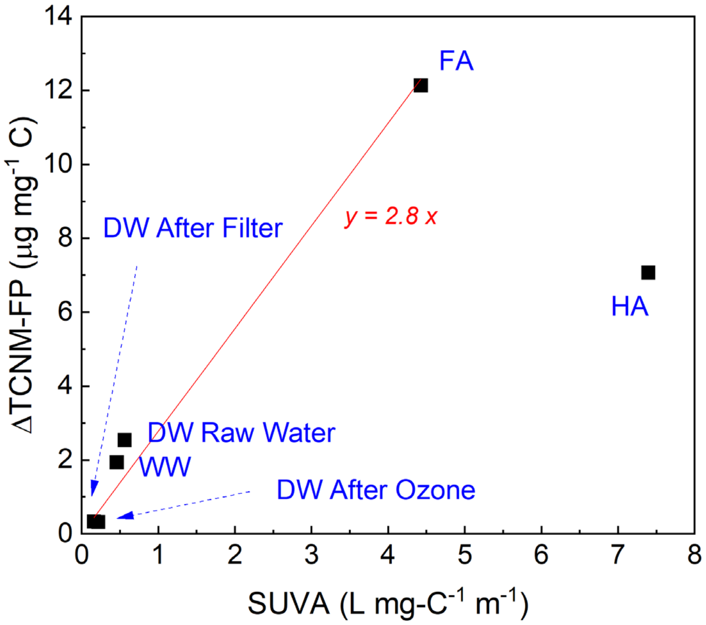

Figure S18. Relationship between the increase of TOC-normalized TCNM-FP after irradiation by 100 mJ·cm^{-2} at 222 nm and SUVA for HA, FA, WW, and DW samples. Conditions: 3.5 mg-C·L^{-1} TOC and 10 mg-N·L^{-1} nitrate. ΔTCNM-FP = TCNM-FP$_{100\ mJ\cdot cm-2}$ – TCNM-FP$_{0\ mJ\cdot cm-2}$.

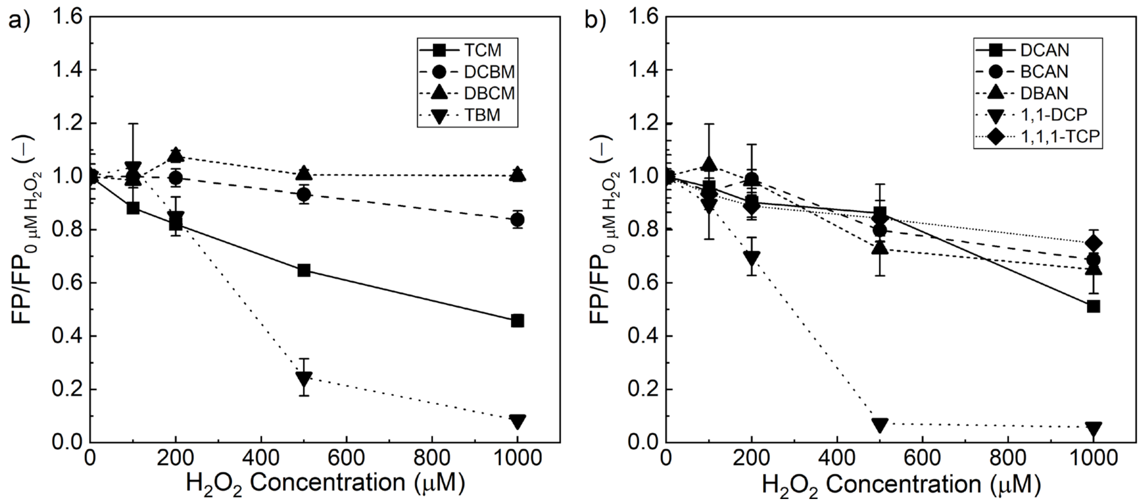

Figure S19. Change of FP for THMs, HANs and HKs after irradiation at 222 nm for HA solution with respect to H_2O_2 concentration at a fluence of 500 mJ·cm^{-2}. Conditions: 3.5 mg-C·L^{-1} TOC, 10 mg-N·L^{-1} nitrate, 100 mg·L^{-1} chloride, 0.1 mg·L^{-1} bromide, and pH 6.8 by 10 mM phosphate.

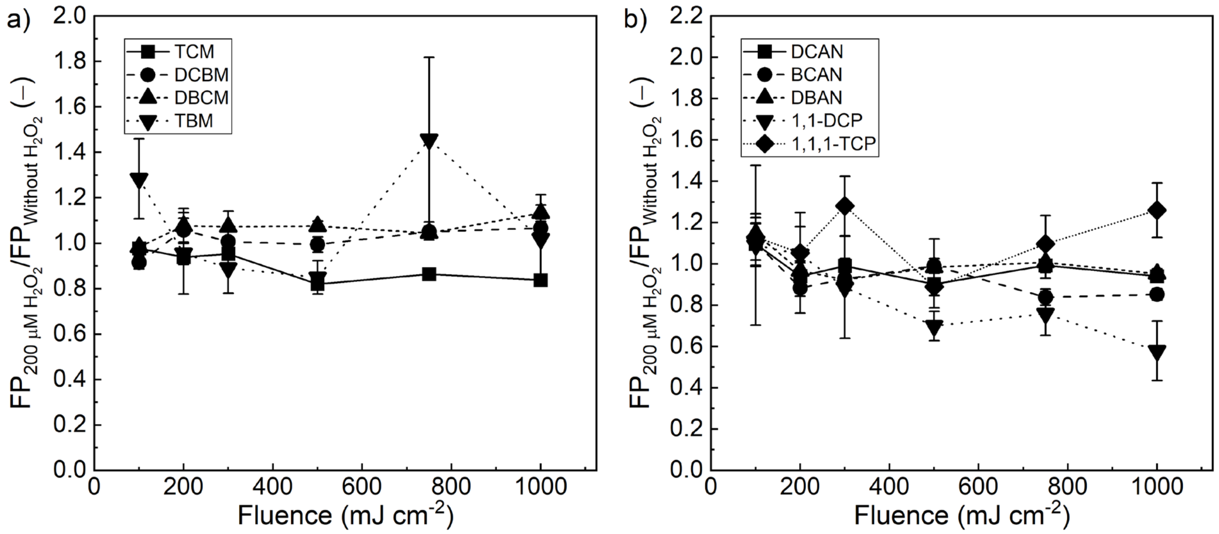

Figure S20. Change of FP for THMs, HANs and HKs after irradiation at 222 nm for HA solution with respect to irradiation fluence in the spike of 200 μM H_2O_2. Conditions: 3.5 mg-C·L^{-1} TOC, 10 mg-N·L^{-1} nitrate, 100 mg·L^{-1} chloride, 0.1 mg·L^{-1} bromide, and pH 6.8 by 10 mM phosphate.

References

(1) Krasner, S. W.; Westerhoff, P.; Chen, B.; Rittmann, B. E.; Nam, S.-N.; Amy, G. Impact of Wastewater Treatment Processes on Organic Carbon, Organic Nitrogen, and Dbp Precursors in Effluent Organic Matter. *Environ Sci Technol* **2009**, *43* (8), 2911-2918.

(2) Xu, J.; Kralles, Z. T.; Dai, N. Effects of Sunlight on the Trichloronitromethane Formation Potential of Wastewater Effluents: Dependence on Nitrite Concentration. *Environ Sci Technol* **2019**, *53* (8), 4285-4294.

(3) Yu, Y.; Reckhow, D. A. Kinetic Analysis of Haloacetonitrile Stability in Drinking Waters. *Environ Sci Technol* **2015**, *49* (18), 11028-11036. DOI: 10.1021/acs.est.5b02772.

(4) Kim, J.; Huang, C.-H. Reactivity of Peracetic Acid with Organic Compounds: A Critical Review. *ACS ES&T Water* **2021**, *1* (1), 15-33. DOI: 10.1021/acsestwater.0c00029.

(5) Bolton, J. R.; Mayor-Smith, I.; Linden, K. G. Rethinking the Concepts of Fluence (Uv Dose) and Fluence Rate: The Importance of Photon-Based Units – a Systemic Review. *Photochemistry and Photobiology* **2015**, *91* (6), 1252-1262, https://doi.org/10.1111/php.12512. DOI: https://doi.org/10.1111/php.12512 (acccessed 2023/07/11).

(6) Payne, E. M.; Liu, B.; Mullen, L.; Linden, K. G. Uv 222 Nm Emission from Krcl* Excimer Lamps Greatly Improves Advanced Oxidation Performance in Water Treatment. *Environ Sci Tech Let* **2022**, *9* (9), 779-785. DOI: 10.1021/acs.estlett.2c00472.

(7) Wang, Y.; Ma, B.; He, C.; Xia, D.; Yin, R. Nitrate Protects Microorganisms and Promotes Formation of Toxic Nitrogenous Byproducts During Water Disinfection by Far-Uvc Radiation. *Environ Sci Technol* **2023**, *57* (24), 9064-9074. DOI: 10.1021/acs.est.3c00824.
